# Zebrafish lacking functional DNA polymerase gamma survive to juvenile stage, despite rapid and sustained mitochondrial DNA depletion, altered energetics and growth

**DOI:** 10.1093/nar/gkv1139

**Published:** 2015-10-30

**Authors:** Jennifer J. Rahn, Jennifer E. Bestman, Krista D. Stackley, Sherine S.L. Chan

**Affiliations:** Department of Drug Discovery and Biomedical Sciences, Medical University of South Carolina, Charleston, SC 29425, USA

## Abstract

DNA polymerase gamma (POLG) is essential for replication and repair of mitochondrial DNA (mtDNA). Mutations in POLG cause mtDNA instability and a diverse range of poorly understood human diseases. Here, we created a unique Polg animal model, by modifying *polg* within the critical and highly conserved polymerase domain in zebrafish. *polg^+/−^* offspring were indistinguishable from WT siblings in multiple phenotypic and biochemical measures. However, *polg^−/−^* mutants developed severe mtDNA depletion by one week post-fertilization (wpf), developed slowly and had regenerative defects, yet surprisingly survived up to 4 wpf. An *in vivo* mtDNA polymerase activity assay utilizing ethidium bromide (EtBr) to deplete mtDNA, showed that *polg^+/−^* and WT zebrafish fully recover mtDNA content two weeks post-EtBr removal. EtBr further reduced already low levels of mtDNA in *polg^−/−^* animals, but mtDNA content did not recover following release from EtBr. Despite significantly decreased respiration that corresponded with tissue-specific levels of mtDNA, *polg^−/−^* animals had WT levels of ATP and no increase in lactate. This zebrafish model of mitochondrial disease now provides unique opportunities for studying mtDNA instability from multiple angles, as *polg^−/−^* mutants can survive to juvenile stage, rather than lose viability in embryogenesis as seen in Polg mutant mice.

## INTRODUCTION

DNA polymerase gamma (POLG) is the sole mtDNA polymerase in vertebrates, and is absolutely required for mtDNA replication and repair (1). As the majority of mtDNA mutations occur through POLG-mediated mtDNA replication errors (2) and since the mitochondrial genome encodes essential elements for oxidative phosphorylation (OXPHOS), the end result is often disrupted energy production (3). *POLG*, a nuclear gene, is a major locus for human mitochondrial disease, and the carrier rate for *POLG* mutations is greater than 2% (4). There are over 200 pathogenic *POLG* mutations linked to a diverse range of disorders that vary in severity, age of onset, and affected tissues (4). Most POLG disorders are accompanied by some degree of mtDNA depletion or mutations, but abnormalities can differ among patients and even among tissues within the same patient (5). In fact, symptoms can vary greatly between patients with the same *POLG* genotype (6). Such heterogeneity makes diagnosis difficult (7,8), and unfortunately there are no effective treatments currently available for patients suffering from these diseases (9).

In order to understand the relationship between *POLG* mutations and disease outcome, several invertebrate model systems have been employed including yeast (10), *Drosophila* (11,12), and *C. elegans* (13). Functional knockout of *POLG* in vertebrates such as mice causes nearly complete loss of mtDNA and lethality at embryonic day 7.5–8.5, while heterozygous animals retain normal mtDNA content and phenotype (14). The ‘mutator mouse’ harboring a D257A knock-in transgene inactivating the exonuclease function ages prematurely due to clonal expansion of mtDNA deletion and point mutations (15–17), and mice carrying the D181A heart-specific (18), D181A brain-specific (19), and Y955C heart-specific (20) mutations develop mtDNA point mutations, deletions, or depletion within those tissues. However, new models are needed to address the progression of POLG mitochondrial disease, especially during early development, as many POLG-related mitochondrial diseases occur in childhood (4).

**Figure 1.**
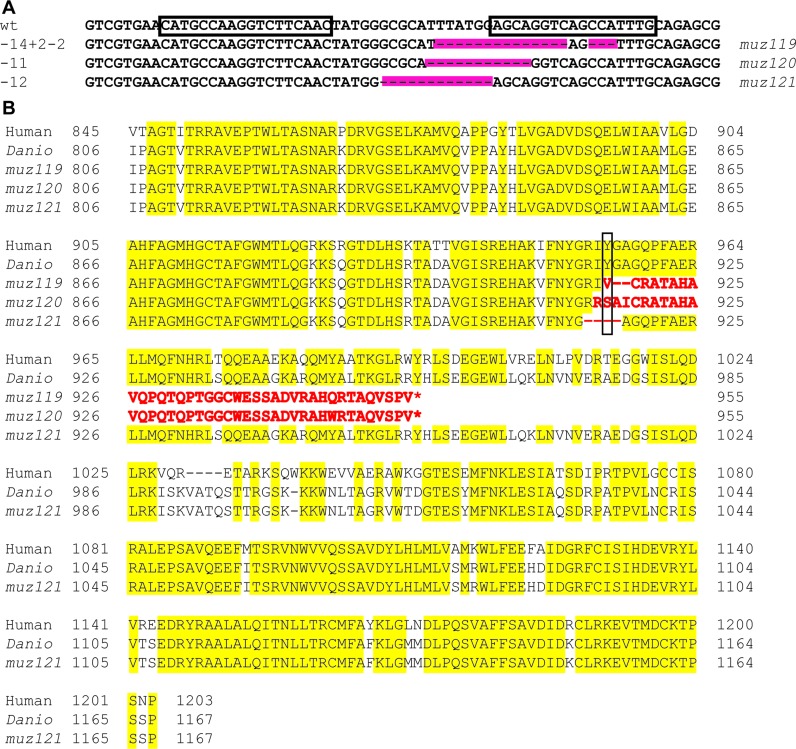
TALEN-induced mutations in *Danio rerio polg*. (**A**) Sequence alignment of TALEN region. TALEN binding sites are shown (boxes). Three different mutations were identified. Alleles were named *muz119*, *muz120*, *muz121*. (**B**) Protein alignment of human, WT *Danio*, and three identified mutant alleles of the polymerase domain of Polg. Two mutant alleles (*muz119* and *muz120*) yield proteins with similar miscoding and a premature stop. The *muz121* allele results in a four amino acid deletion spanning hY955 (boxed).

Zebrafish have proven to be excellent vertebrate model organisms for studying developmental processes as well as progression of disease, due in part to the ease of *in vivo* manipulation (21). Zebrafish are also advantageous for *in vivo* drug screening (22). Thus, we have specifically developed new methodologies and approaches for studying mitochondrial dysfunction *in vivo* in zebrafish (23–26). In this study, we have used TALEN technology to generate three stable lines carrying mutations spanning amino acid hY955 (dY916) in Polg's polymerase domain (Figure 1B). We found that *polg*^−/−^ zebrafish survived up to the juvenile stage despite severe and sustained mtDNA depletion that occurs just after embryogenesis. These mutants also revealed tissue-specific mtDNA depletion, reflecting the tissue differences found in human POLG mitochondrial disease. Our model now permits detailed study of why some tissues are more vulnerable to compromises in mtDNA content and respiratory output than others. Insights into disease progression and the mechanisms inherent in zebrafish that allow these animals to survive with severe mtDNA depletion are key advantages for using this model to develop effective treatment strategies for mitochondrial diseases.

## MATERIALS AND METHODS

### Zebrafish husbandry and generation of *polg* mutant lines

Zebrafish were crossed according to standard methods and embryos were raised to juvenile stage according to Westerfield *et al*. (27). Fertilized eggs were collected and placed in egg water, and maintained in an incubator set at 28.5°C with a 14/10 h light/dark cycle. Starting at 7 days post-fertilization (dpf), animals were fed a standard diet of paramecia and plankton. All animal studies were approved by the Medical University of South Carolina Institutional Animal Care and Use Committee (AR #2850) and performed in accordance with the guidelines.

TALEN vectors targeted to *polg* were produced by Dr Timothy Dahlem, Mutation Generation and Detection Core, University of Utah (Supplemental Figure S1, (28)). Each vector was linearized with NotI (New England Biolabs) and purified before being used to generate mRNA with an SP6 in vitro transcription reaction (mMessage mMachine kit Ambion/Invitrogen). A mixture of 500 ng left TALEN (DD) and right TALEN (RR) mRNA was made with 1 μl phenol red at 7 μl dH_2_O and was injected into 1–4 cell stage zebrafish embryos at 1 nl per embryo. After confirmation of TALEN function by screening 24 injected embryos by high resolution melt analysis (HRM, see below), embryos were grown to adulthood. At three months of age, each fish was outcrossed to WT AB fish and 24 embryos screened by HRM to identify fish that carried *polg* germline mutations (Supplementary Figure S2). Those TALEN-injected fish that produced mutant embryos as a result of this outcross were termed founders (G0). Embryos from these outcrosses were grown to adulthood and screened by tail clip at three months of age. Fish that tested positive by this analysis were the F1 generation bearing the mutation in all cells and were kept as the breeding population. Material from representative fish for each HRM curve was cloned (TA cloning kit, Invitrogen, Carlsbad, CA, USA) and sequenced for identification of the mutation.

### High resolution melt analysis (HRM)

Fish were screened for presence of a *polg* mutation using either whole embryos or tail clips performed at 3–28 dpf or tail clips from adult fish using an HRM technique modified from Dahlem *et al*. (28). Material was digested in 30–50 μl DNA extraction buffer (10 mM Tris–HCl pH 8.0, 1 mM EDTA, 50 mM KCl, 0.3% Tween-20, 0.3% NP-40, 500 μg/ml proteinase K) at 55°C, for 2 h followed by inactivation at 99°C for 5 min. Samples were incubated overnight at 4°C. Reactions were set up in duplicate for each sample containing 5 μl 2× SsoFast EvaGreen Supermix (Bio-Rad), 0.25 μl each 10 μM forward (F) and reverse (R) primers (F: GTCGTGAACATGCCAAGGTCT; R: TGTGGTTGAACTGCATGAGC), 1 μl DNA solution, and 3.5 μl H_2_O. Amplification was performed using a CFX-96 (Bio-Rad) instrument and the following program: 95°C 3 min, 40 cycles of 95°C for 5 s, 60°C 5 s followed by a melt program starting at 65°C for 10 s and increasing 0.2°C every step up to 95°C. Data was plotted using Bio-Rad CFX Manager software and melt curves were compared to WT fish material for each run. Genotype was determined based on comparison to curves generated from cloned and sequenced material (Supplemental Figure S3).

### Tail clipping for genotyping and quantification of fish growth and caudal fin regeneration

Zebrafish between 3 dpf and 3 weeks post-fertilization (wpf) were anesthetized in 0.02% Tris-buffered Tricaine and then placed on a glass slide on a dissecting microscope stage. The caudal fin (tail) was cut distal to the notochord tip with a scalpel blade. Fish were either frozen individually in microcentrifuge tubes to await molecular analyses, or were housed individually in a 24-well tissue culture dish in E2 embryo media with methylene blue (http://zebrafish.org/documents/protocols.php) such that caudal fin regeneration and growth could be quantified. Fish were examined and photographed using a Zeiss Axio Observer A1 microscope and AxioCam HRc CCD camera. Growth metrics were conducted as outlined in (29) and all measurements were obtained with the FIJI image processing package of ImageJ (30). Regeneration levels were determined by comparing images taken immediately and 3 days after amputation of the fin. Statistical analyses were conducted using JMP 11 (SAS) and unless noted, Mann–Whitney unpaired tests were used.

### Survival analysis

Survival of *polg*^+/+^, *polg*^+/−^ and *polg*^−/−^ zebrafish was determined by comparing observed genotype ratios to the expected ratio of 25:50:25. Fertilized eggs were collected from individual spawning events over several months of breeding and reared in beakers. Individuals were collected over the course of the study period (3 dpf to 3 wpf) and were genotyped using the HRM method either on extracted DNA or tail clip material. Once genotype was determined, data from each age was combined and genotype proportion was determined. Pearson's Chi-square analyses were used to test whether expected genotype proportions were recovered at each time point.

### MtDNA content analysis of whole larvae and sections

Single animals (1–4 wpf) were incubated overnight at 55°C in 250 μl digestion solution (50 mM Tris–HCl pH 8.0, 200 mM NaCl, 100 mM EDTA pH 8.0, 1% SDS, 0.2% DTT, 2 mg/ml proteinase K). DNA was prepared using phenol chloroform extraction (31) and quantified by PicoGreen assay (Invitrogen, Grand Island, NY, USA). Single embryos at 3 dpf, and individual or grouped organs, were digested in HRM buffer (see above) and not subjected to further extraction before being quantified by PicoGreen assay as above. 8.5 ng of DNA was used in a qPCR reaction using SsoAdvanced SYBR Green Supermix (BioRad, Hercules, CA, USA) with appropriate primers (300 mM each) using the BioRad CFX96 RealTime instrument as previously described (23). Each sample was run in duplicate and a mean PCR cycle threshold (Ct) value generated for *ef1a* and *nd1*. Only samples with *ef1a* Ct values between 22 and 23 were included in this analysis. Delta Ct (dCt) was calculated by subtracting the Ct of *ef1a* from the Ct of *nd1* for each sample. Standard error of the mean calculations and statistical analyses by Mann-Whitney unpaired nonparametric tests were performed using JMP.

### Western blot analysis

Groups of 10 frozen genotyped larvae frozen at 3 wpf were subjected to mitochondrial fractionation using a Mitochondrial Isolation Kit for Tissues (Abcam) following manufacturer's instructions. Pellets of enriched mitochondria were resuspended in 50 μl Laemmli Sample buffer (Bio-Rad) with 2.5 μl β-mercaptoethanol and sonicated for 5 s on ice. Equal volumes of crude fractionate were loaded on 7.5% (for Polg) or 12% (for Vdac1) Bio-Rad MiniProtean TGX polyacrylamide gels and subjected to SDS-PAGE at 75 V for 90 min. Recombinant human POLG protein was produced and run alongside zebrafish mitochondrial samples as a positive control for the Polg blot (32). Gels were transferred to PVDF (Millipore) with 25 mM Tris, 195 mM Glycine transfer buffer using a Bio-Rad submerged transfer apparatus. Blots were then stained using MemCode reagent (Pierce) and photographed before being cleared as per the manufacturer's protocol. Blocking was performed for 1 h at room temperature in 2.5% non-fat dry milk in Tris-buffered saline + Tween 20 (TBST) for Polg and 5% non-fat dry milk in TBST for Vdac1. The custom Polg antibody was made in rabbits against a synthetic peptide (N-EITKGSLTKEKRQAPRSK-C) located near the C-terminus of the zebrafish Polg (ThermoFisher) and bleeds were affinity purified before use in western blots at 1:300 dilution. Vdac1 polyclonal antibody was obtained from Abcam (ab15895) and used at 1:1000 dilution. All primary incubations were performed at 4°C overnight. Washes were done 3 × 10 min with TBST followed by secondary antibody (goat anti-rabbit or rabbit anti-mouse HRP, Bio-Rad) incubations in the same block buffer at 1:10 000 dilutions for 1 h at room temperature. Washes were repeated, and blots exposed to Clarity ECL solution (Bio-Rad) and imaged using the ImageQuant instrument (GE). Densitometry was performed using FIJI and signal intensity was normalized to MemCode staining (Pierce).

### *In vivo* mtDNA polymerase activity assay

Large numbers (200–400) of 3–5 h post-fertilization (hpf) embryos were exposed to 0 or 15 μg/ml ethidium bromide (EtBr, EMD chemicals) in 30 ml standard embryo media in 100 cm petri dishes for 6 days. At 72 hpf, media was removed and fresh embryo media with or without EtBr was added. After 6 days, all media was removed and larvae were washed three times with fresh embryo media and larvae were reared in individual beakers prior to harvest at 1, 2 and 3 wpf for mtDNA content analysis.

### ATP and lactate assays

Individual genotyped larvae were processed for ATP using the ATP Fluorometric Assay Kit (Biovision K354-100) as per our previous studies (23). L-lactate levels were determined using a modification of the method of Brandt *et al*. (33), where fluorescence of NADH generated from oxidation of lactate to pyruvate via lactate dehydrogenase is measured. All chemicals were obtained from Sigma unless otherwise noted. Briefly, snap-frozen larvae were homogenized in 75 μl Triton X-100 buffer (1% Triton X-100, 150 mM NaCl, 50 mM Tris–HCl pH 7.2) and incubated on ice for 10 min. Samples were centrifuged at 13 000 g for 10 min at 4°C, and the supernatant retained. l-(+)-Lactic acid was serially diluted (3.125–25 μM) and added to each plate for the standard curve. For all samples, a 1:10 dilution was made and L-lactate measured by combining 20 μl sample (or standard) with 96 μl embryo media, 63 μl 1 M glycine pH 8.3, 6 μl 1 M hydrazine, 10 μl 20 mM NAD^+^ and 5 μl 84.91 units/ml lactate dehydrogenase in a multi-well plate. Plates were incubated at 37°C for 30 min and fluorescence measured with excitation of 355 nm; emission 460 nm in a Tecan plate reader. Protein concentrations of lysate were determined by BCA assay (Sigma) and concentration calculated by comparison to a standard curve. Total ATP or lactate was normalized to μg protein per well.

### Tissue respiration

Oxygen consumption rate (OCR) measurements from zebrafish tissue were performed using the XF-24 Extracellular Flux Analyzer (Seahorse Bioscience) using standard methods (26). Central nervous system (CNS) tissue was dissected from anesthetized fish and bathed in 700 μl Evans Saline (34) in a 24-well islet plate and kept in place with an islet capture screen. Basal respiration was calculated for each animal from the two consecutive OCR readings before 2 μM carbonyl cyanide-4-(trifluoromethoxy)phenylhydrazone (FCCP) was injected into each well. FCCP-induced responses were determined by averaging the maximum OCR after injection. To eliminate differences caused by the variation in the size of the animals, rates were normalized to the eye size of the fish. As the CNS includes the eyes and was the tissue assayed, eye size was used to normalize OCR. One way ANOVA/Steel post hoc tests were conducted to determine significant differences between the groups.

## RESULTS

### Engineering *polg* mutant zebrafish

Human POLG is 1239 amino acids (aa) in length and is composed of three major domains: exonuclease domain (171–440), linker region (476–785), and polymerase domain (816–1239) (Supplemental Figure S1; http://tools.niehs.nih.gov/polg/). The *Danio rerio* homologue is 1206aa in length with the same functional domains (Figure 1B) and a 69% overall identity (79% similarity) to human POLG (35). We generated mutations in the polymerase domain using custom TALEN vectors (Supplemental Figure S1) targeting the Y916 amino acid, equivalent to position Y955 in humans (Figure 1A and B). In humans, mutation to cytosine at position 955 (Y955C) results in development of multiple disease phenotypes including progressive external ophthalmoplegia and parkinsonism (36,37). Biochemical studies show that POLG Y955C has poor polymerase activity and fidelity, highlighting the critical nature of this region (38).

Three different *polg* mutant lines were generated by TALEN gene editing (Figure 1A and Supplemental Figure S1). Two mutant alleles (*polg^muz119^* and *polg^muz120^*) translate into proteins differing only by 2aa in the nonsense stretch. The *polg^muz119^* allele is a 14+2–2 bp mutation (Figure 1A) resulting in 38aa of miscoded protein sequence after aa916 and a premature stop at aa955 (Figure 1B). Similarly, the *polg^muz120^* allele has an -11bp deletion (Figure 1A) producing a 41aa miscoded protein sequence after aa917, resulting in a premature stop at aa955 (Figure 1B). The *polg^muz121^* allele has a -12bp deletion, yielding a protein with only 4aa removed, including the critical dY916, while retaining the reading frame and all additional coding sequence (Figure 1A, B).

We generated a breeding population of F1 heterozygous mutant zebrafish termed *polg^+/muz119^*, *polg^+/muz120^*, *polg^+/muz121^*. All genotypes, with the exception of *polg^muz120/muz120^*, are easily distinguished by high resolution melt (HRM) analysis of DNA obtained by fin clips (Supplemental Figure S2). In this study we focused primarily on mutant alleles *polg^muz119^* and *polg^muz120^*, which result in truncation of Polg protein in the polymerase domain. For clarity, data from *polg^+/muz119^* and *polg^+/muz120^* were combined and presented as *polg^+/−^* while *polg^muz119/muz119^*, *polg^muz120/muz120^*, and *polg^muz119/muz120^* are presented as *polg^−/−^*. Data for individual genotypes are presented in supplemental material where appropriate.

### Most *polg*^−/−^ animals fail to survive to four weeks

**Figure 2.**
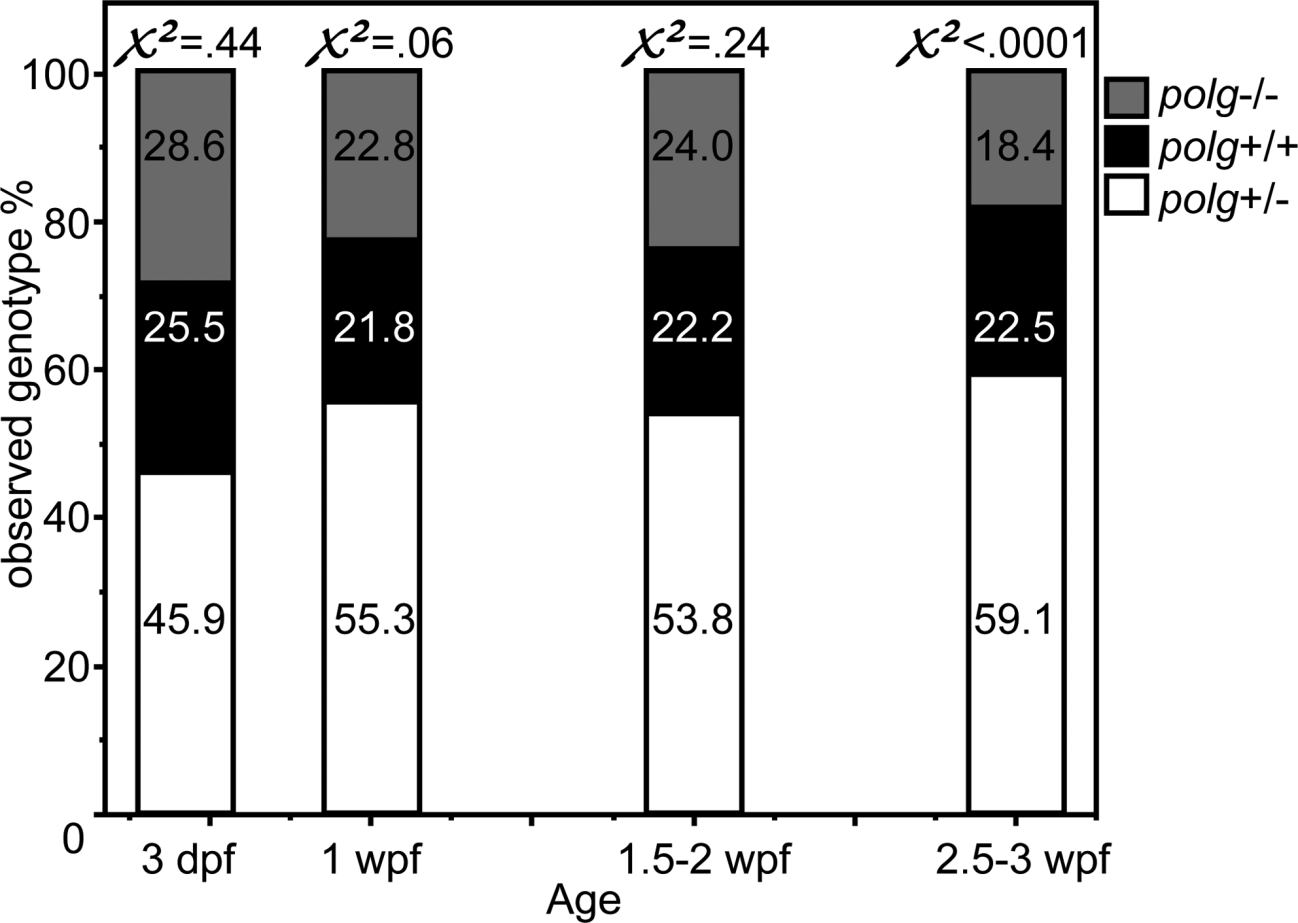
*polg^−/−^* zebrafish exhibit decreased survival by 3 wpf. Proportions of each genotype are shown for each age range of zebrafish. Pearson's Chi-square values are shown above each bar indicating whether the observed ratios were significantly different from the expected ratios. *N* = 196 for 3 dpf, 486 for 1 wpf, 433 for 1.5–2 wpf, and 875 for 2.5–3 wpf.

We crossed *polg^+/−^* animals over many weeks and used HRM to genotype offspring that were collected for mtDNA analysis and other experiments. Interbreeding F1 heterozygous fish generated predictable proportions of *polg^+/+^*, *polg^+/−^* and *polg^−/−^* (25%, 50%, 25%). The observed proportions did not significantly differ from predicted until 2.5–3 wpf when 22.5% *polg^+/+^*, 59.1% *polg^+/−^* and 18.4% *polg^−/−^* were recovered. This distribution of progeny was significantly different from the expected ratios (*χ*^2^ < 0.0001; Figure 2). These data indicate that *polg^−/−^* animals have normal survival until 2 wpf, but begin to die after that time, such that fewer than expected were recovered by 3 wpf. Zebrafish reared in our laboratory typically survive up to 2–3 years of age.

### MtDNA is depleted early in *polg^−/−^* animals but remains unchanged in *polg^+/−^* animals

**Figure 3.**
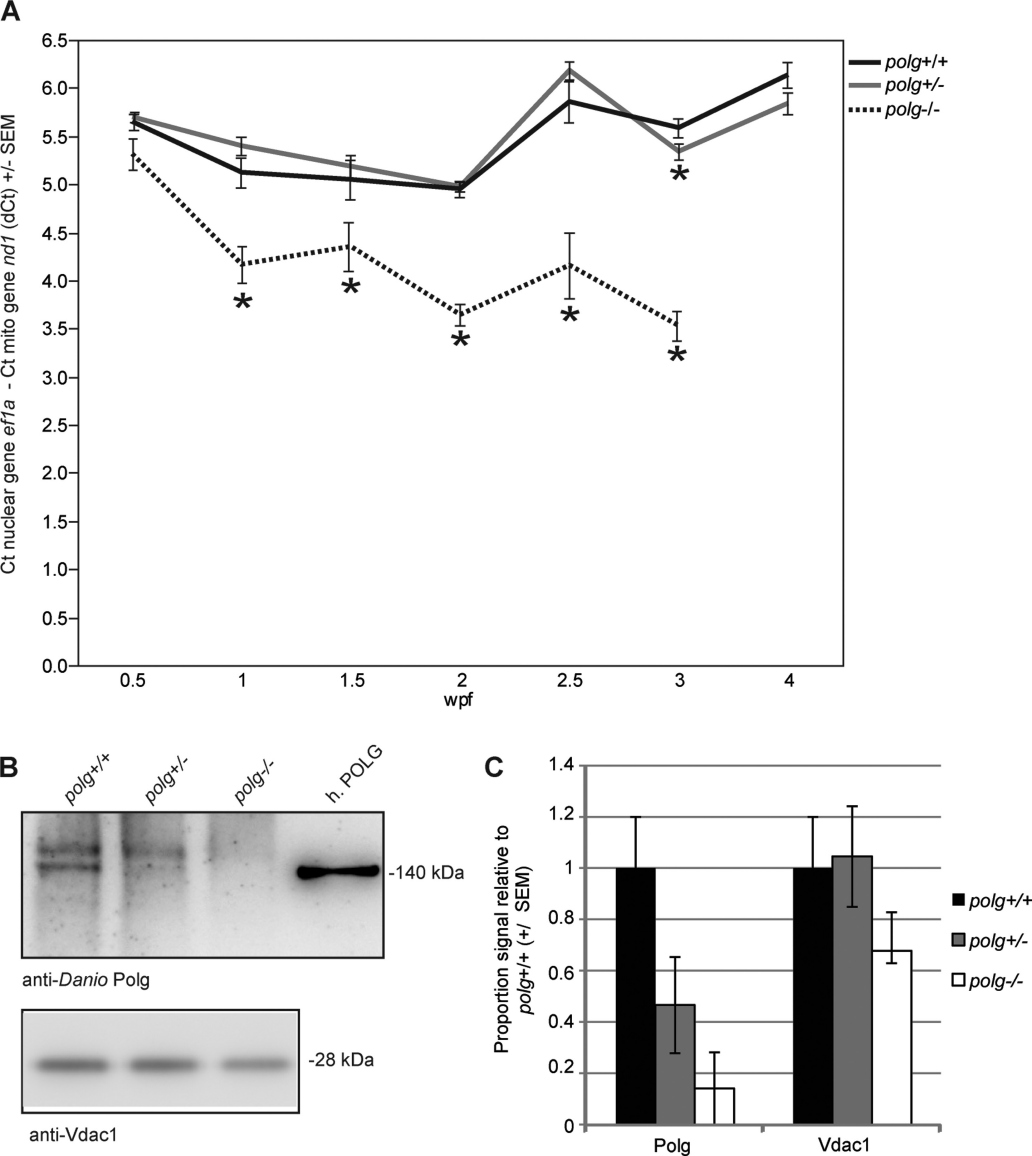
*polg^−/−^* zebrafish exhibit severe and sustained mtDNA depletion, have less full-length Polg and Vdac1 protein while heterozygous zebrafish have similar levels as WT. (**A**) DNA was isolated from whole individuals at various time points. Mean dCt values were calculated as Ct of *nd1* (mitochondrially-encoded gene) minus Ct of *ef1a* (nuclear gene) and plotted with SEM (see Supplemental Table S1 for dCt values, sample sizes for each time point and *P*-values). * indicates *P* ≤ 0.05 difference from WT by Mann–Whitney nonparametric test. (**B**) Representative western blot analysis of Polg and Vdac1 mitochondrial protein from 3 wpf zebrafish larvae. Recombinant human POLG was run on the same gel, as a positive control for the mtDNA polymerase. (**C**) Densitometry of multiple blots. *n* = 3–4.

Because mutations in Polg resulting in deficient polymerase activity can result in mtDNA depletion (39), we hypothesized that qPCR analysis of mtDNA content from *polg^−/−^* zebrafish would reveal a gradual decrease in mtDNA until their death at 3 wpf. Equal amounts of purified DNA were used in order to make comparisons about mtDNA levels between animals and time points, and the difference in Cts for mtDNA and nuclear DNA were calculated to give the dCt. We chose to present dCt values rather than transformed fold change as statistical analyses were performed on the dCt values themselves. Mean mtDNA copy number per cell (calculated by 2^dCt^ then multiplied by 2 to account for two copies of nuclear gene per cell) is reported in the text for comparison purposes. MtDNA levels of WT and heterozygous fish were indistinguishable with the exception of the 3 week time point, where *polg^+/−^* fish had a 15% smaller dCt value than WT with a mean mtDNA copy number per cell of 96 for WT and 81 for *polg^+/−^* fish (*P* < 0.01; Figure 3A, Supplemental Table S1, Figure S3). Overall these data suggest that *polg^+/−^* zebrafish have sufficient Polg protein to sustain mtDNA content. At the first time point (3 dpf), mtDNA levels of *polg^−/−^* animals were not different from WT (*P* = 0.06, Figure 3A, Supplemental Table S1, Supplemental Figure S3). Yet, just 3–4 days later (1 wpf), *polg*^−/−^ fish exhibited significantly lower mtDNA levels with a mean mtDNA copy number per cell of 36, which is only 52% of the WT level (69.6 mtDNA copies per cell) (Figure 3A, Supplemental Table S1, Supplemental Figure S3). MtDNA levels remained severely depleted in these *polg*^−/−^ fish until their death with mean mtDNA copy number per cell reaching a low of 23.2 compared to 96 in WT fish at 3 wpf (24% of WT levels). These data indicate that fish carrying two mutant *polg* alleles were defective for mtDNA replication, resulting in a rapid and sustained depletion of mtDNA content within 1 week. However, despite this early and severe mtDNA depletion, this low level of mtDNA is adequate for fish to survive up to 1 month. Additionally, we assayed the genomic DNA obtained for this analysis for large mtDNA deletions in fish of all genotypes using long-range PCR methods but did not observe any differences in the presence of large deletions among genotypes (Supplemental Figure S4).

We examined Polg protein levels from whole larvae at 3 wpf using an antibody specific for the C-terminus of zebrafish Polg (aa1186–1203). We detected two bands using this antibody: a 170 kDa non-specific band and a 135 kDa band corresponding to the zebrafish Polg protein (Supplemental Figure S1). A sample of recombinant human Polg (140 kDa) was used as a positive control (Figure 3B). This western blot analysis revealed that at 3 wpf, *polg*^+/−^ animals had approximately half the Polg protein compared to WT, while *polg*^−/−^ animals had little detectable protein (Figure 3B and C). To verify that depleted mtDNA correlated with decreased mitochondrial mass, we also examined levels of Vdac1, a protein found on the outer mitochondrial membrane (Figure 3B and C). Heterozygous animals had similar levels of Vdac1 protein as WT, but Vdac1 levels in *polg*^−/−^ animals were 32% lower than WT (Figure 3B). These data indicate that *polg*^−/−^ animals not only lose mtDNA content by 3 wpf, but also have lower mitochondrial mass.

### *polg^−/−^* animals grow more slowly than WT or *polg^+/−^* animals

**Figure 4.**
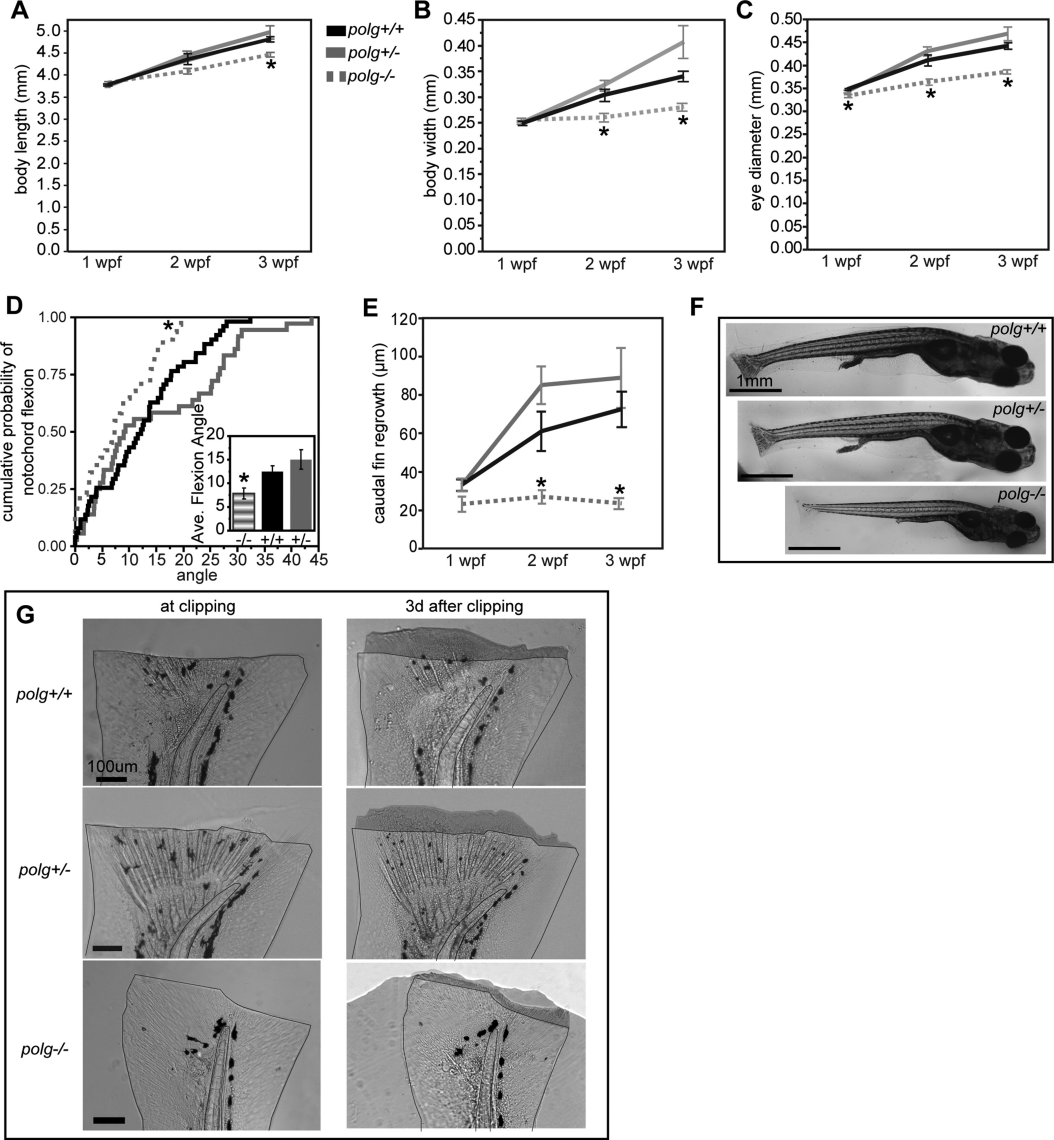
Growth, development, and caudal fin regrowth are altered in *polg^−/−^* but not *polg^+/−^* zebrafish. Means ± SEM of body length (**A**), body width (**B**), and eye diameter (**C**) measured at 1–3 wpf for each genotype. (**D**) The cumulative probability of the angle of notochord flexion at 3 wpf for each genotype with means ± SEM given in the inset. (**E**) Means ± SEM of caudal fin regrowth 3 days after amputation for each age group. (**F**) Images of each zebrafish genotyped at 3 wpf. Scale bar = 1 mm. (**G**) Images of caudal fins from 3 wpf zebrafish immediately after (left column) and 3 days after amputation (right column). Amount of regrowth is shaded. Scale bar = 100 μm. * indicates significant difference from WT; *P* ≤ 0.05 by ANOVA/Steel post hoc test. See Supplemental Table S2 for values, sample sizes and *P*-values.

We quantified the growth characteristics of WT, *polg^+/−^* and *polg^−/−^* animals over the first 3 weeks of development. Zebrafish embryonic development ends at 6 dpf, at which point all major organs have differentiated (40) and the freely swimming larvae hunt for food (41). Larval length from snout to where the caudal fin joins the tail is a quantifiable indicator of normal development in zebrafish (29). *polg^+/−^* mutants exhibited similar growth rates to WT at each time point (Figure 4A, Supplemental Table S2, Supplemental Figure S5). In contrast, *polg^−/−^* animals grew more slowly (Figure 4A, F, Supplemental Table S2, Supplemental Figure S5) and were significantly smaller than WT animals at 3 wpf.

We also measured body width just anterior to the anal fin, a metric that increases proportionally with length (29). Average body widths of heterozygous animals were not significantly different than WT at any time point (Figure 4B, F, Supplemental Table S2, Supplemental Figure S5), nor were the *polg^−/−^* body widths different at 1 wpf. However, *polg^−/−^* animals had significantly smaller widths at 2 and 3 wpf. Calculating the width as a percent of the animal's length at 3 wpf revealed that WT animals (7.0 ± 0.14%, *n* = 49) and heterozygotes (7.8 ± 0.36%, *n* = 30) were not significantly different (*P* = 0.23), but with their width at just 6.2 ± 0.13% their length, the *polg^−/−^* animals were significantly (*P* < 0.01) and disproportionately thin for their length.

Many human disorders associated with *POLG* mutation affect function of the CNS, which includes the eye (42). While WT and *polg^+/−^* zebrafish showed a normal increase in the rostral caudal diameter of the eye throughout development (Figure 4C and F, Supplemental Table S2, Supplemental Figure S5), *polg^−/−^* animals had significantly smaller eye diameters compared to control animals beginning 1 wpf. Comparing the percent of the eye diameter to the length of the 3 wpf animals revealed that at 9.6 ± 0.15% (*n* = 37) of their length, the heterozygous animals had slightly larger eyes for their body size compared to WT animals (9.2 ± 0.07%, *n* = 58; *P* = 0.05). The eyes of *polg^−/−^* animals were 8.6 ± 0.09% (*n* = 36) of their body length, significantly smaller than control animals. This indicates inhibition of eye development in *polg^−/−^* animals, resulting in smaller and disproportionately sized eyes for their body size.

Notochord flexion, the curvature of the distal tip of the notochord, is a developmental milestone occurring after zebrafish reach ∼4.5 mm (29). Because the largest *polg^−/−^* fish reached only 5.24 mm, we compared flexion angles of WT, *polg^+/−^, polg^−/−^* fish of similar size (4.24–5.24 mm). The mean notochord flexion for WT zebrafish was 12.5 ± 1.3° (*n* = 51, Figure 4D, inset) and *polg^+/−^* animals had a similar (*P* = 0.40) mean angle of 15.0 ± 1.6° (*n* = 36, Figure 4D, inset). However, the average flexion angle of *polg^−/−^* fish was 7.9 ± 1.7° (*n* = 31), which was significantly smaller than WT (*P* = 0.04, Figure 4D, inset). The cumulative probability distribution for *polg^−/−^* animals was significantly shifted from the WT distribution (Kolmogorov–Smirnov test, *P* = 0.04), while *polg^+/−^* animals were not significantly different from WT (*P* = 0.14, Figure 4D). Altogether, these developmental metrics indicate that *polg^−/−^* animals develop more slowly, yielding 3-week old animals that were significantly and disproportionately smaller than heterozygote or WT animals. In contrast, the growth characteristics of heterozygous fish were not significantly different from WT.

### *polg^−/−^* animals have defective caudal fin regeneration

The caudal fins of zebrafish readily and rapidly regenerate (43), making this tissue a common source of DNA for genotyping. Surprisingly, weeks after amputation we noticed that the caudal fins of *polg^−/−^* fish did not grow back and fully regenerate like heterozygote and WT fish. After observing these differences, we sought to quantify the level of tissue regeneration in 1–3 wpf animals, 3 days after amputation. We found that *polg^+/−^* animals had similar levels of regeneration as WT at each time point (Figure 4E and G; Supplemental Table S2). The amount of regeneration of *polg^−/−^* animals was also similar to WT at 1 wpf, however fin regeneration was significantly lower at 2 and 3 wpf. These data indicate that in later stages *polg^−/−^* zebrafish have less regenerative capacity than WT animals of the same age.

### ATP and glycolysis are unchanged in *polg^−/−^* animals despite decreased mitochondrial mass

**Figure 5.**
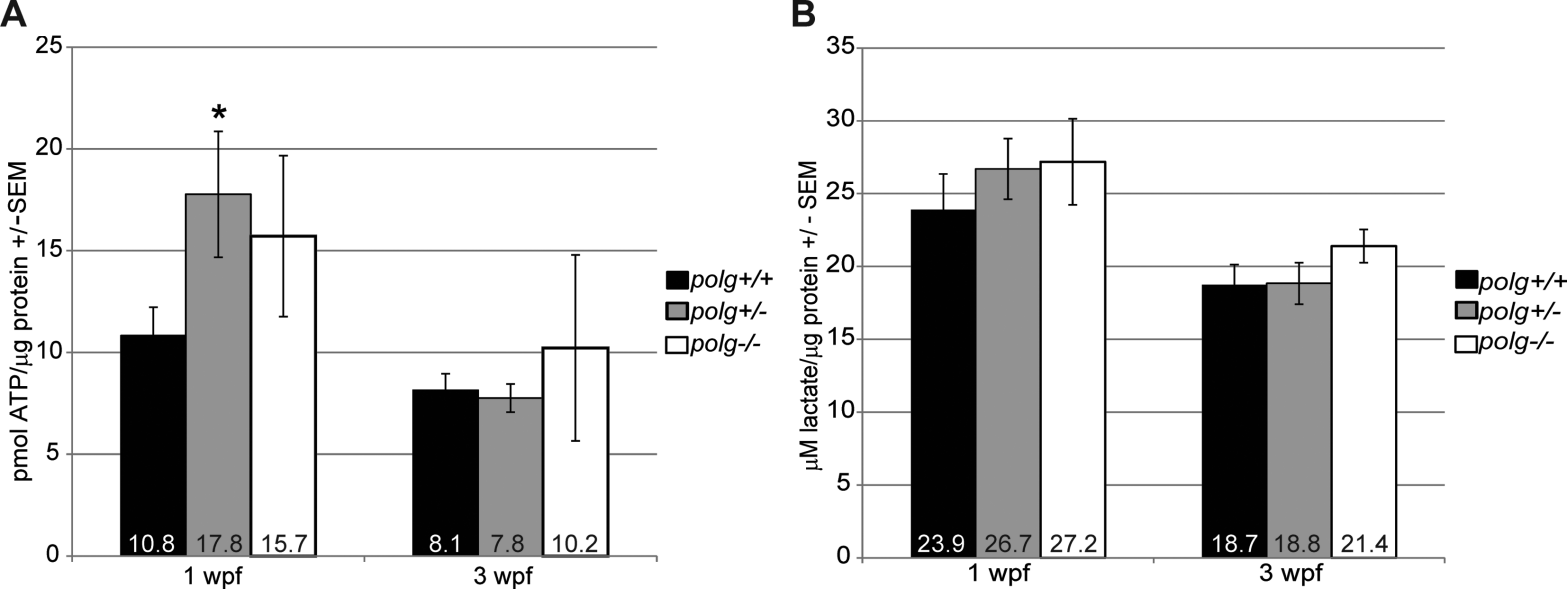
ATP and lactate are not altered in *polg^−/−^* zebrafish. (**A**) Mean whole body ATP content normalized to protein concentration is plotted with SEM for zebrafish at 1 and 3 wpf. (**B**) Whole body lactate normalized to protein concentration is plotted with SEM for zebrafish at 1 and 3 wpf. Mean values are displayed on respective bars. * indicates significant difference from WT; *P* ≤ 0.05 by *t*-test.

Since depletion of mtDNA results in fewer functional OXPHOS complexes and impaired mitochondrial function (44), we predicted that ATP levels would be lower in *polg^−/−^* but unchanged in *polg^+/−^* animals. Surprisingly, at 1 wpf we found that *polg^+/−^* animals had significantly higher ATP levels (*n* = 4–5; *P* = 0.05). Furthermore, ATP levels of *polg^−/−^* animals were indistinguishable from WT (*n* = 3–5, *P* = 0.21, Figure 5A), despite their low mtDNA levels at this time point (Figure 3). At 3 wpf, when many *polg^−/−^* animals begin to die (Figure 2), the ATP levels of both *polg^−/−^* and *polg^+/−^* animals were not significantly different from WT (*n* = 5, *P* = 0.16 and *n* = 5, *P* = 0.75, respectively, Figure 5A).

We next measured static lactate levels in zebrafish to estimate glycolysis, the non-mitochondrial energy producing pathway. Levels of l-lactate (a byproduct of glycolysis) from 10 *polg^−/−^*, 12 *polg^+/−^*, and 10 WT fish were measured at 1 wpf. All *polg* genotypes had similar lactate levels at this time point (*P* = 0.39 and *P* = 0.40, respectively, Figure 5B). Similarly, lactate levels of the 3 wpf fish were also not significantly different between groups (*n* = 15–16, *P* = 0.96 and *P* = 0.15 respectively, Figure 5B). These data suggest that *polg^−/−^* animals do not upregulate the glycolytic pathway to generate ATP.

### *In vivo* mtDNA polymerase activity assay reveals that unlike *polg^+/−^* animals, *polg^−/−^* zebrafish do not recover from mtDNA depletion

To test the polymerase activity of WT and mutant Polg, we developed an *in vivo* mtDNA polymerase activity assay. EtBr is an intercalating dye that preferentially incorporates into circular DNA, halting replication and transcription. It is commonly used to deplete mtDNA to generate ‘rho zero’ cells, with little to no effect on nuclear DNA (45). Treating live fish with EtBr to block mtDNA replication allowed us to assess whether full-length and mutant Polg proteins can repopulate mtDNA after removal of EtBr. We hypothesized that WT animals with fully active Polg would successfully repopulate mtDNA, while those with partially functional Polg may exhibit delayed or decreased repopulation rates.

**Figure 6.**
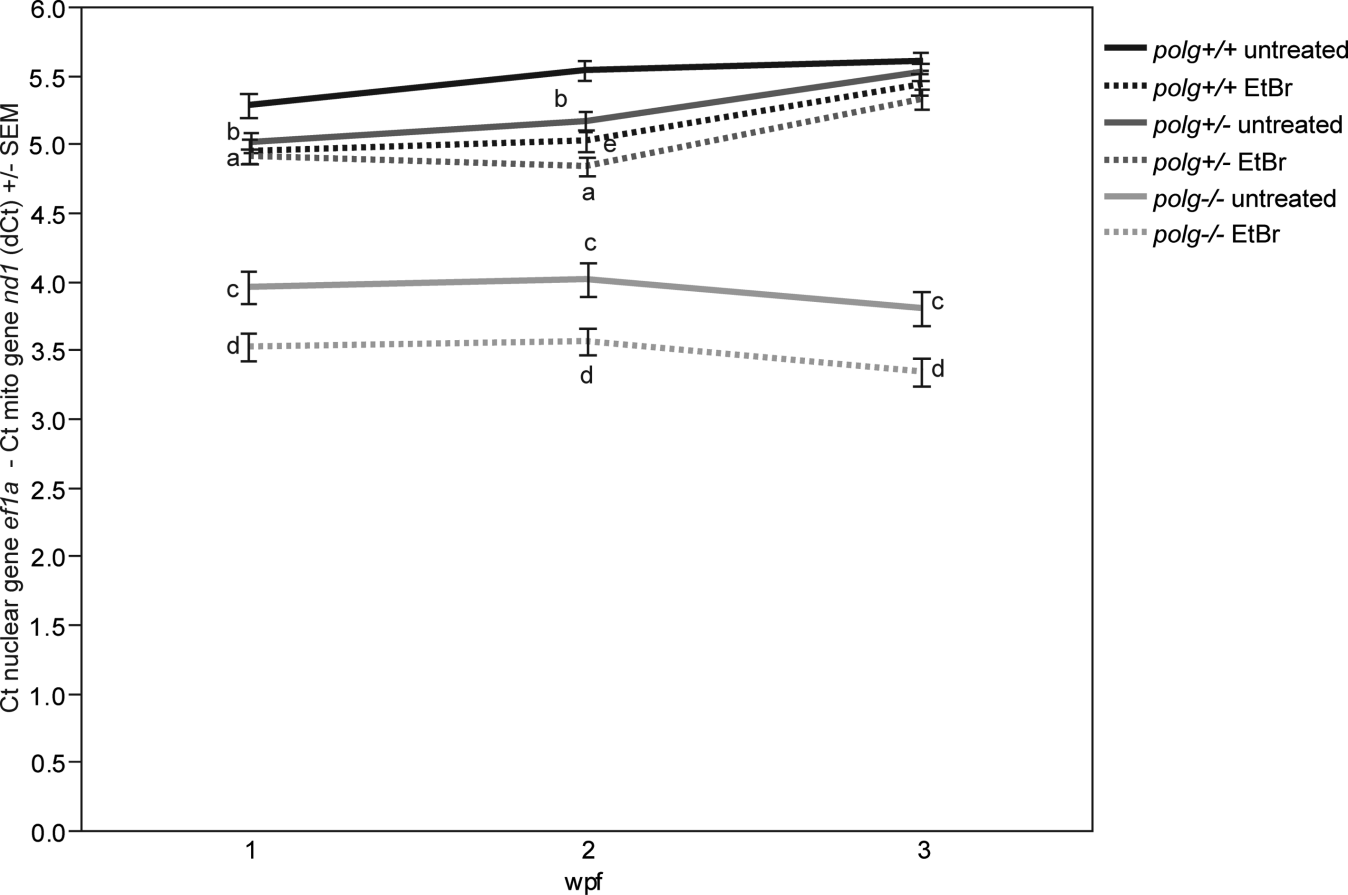
*polg^+/+^* and *polg^+/−^* zebrafish recover from EtBr-induced mtDNA depletion while *polg^−/−^* zebrafish remain depleted of mtDNA. Zebrafish embryos were treated with EtBr starting at 3 hpf for 6 days before EtBr was removed. Mean dCt values at 1, 2 and 3 wpf are shown with SEM (see Supplemental Table S3 for fold change values, sample sizes and *P*-values). (**a**) Indicates significant difference between untreated WT and EtBr-treated WT; (**b**) indicates significant difference between untreated WT and untreated *polg^+/−^*; (**c**) indicates significant difference between untreated WT and untreated *polg^−/−^*; (**d**) indicates significant difference between untreated *polg^−/−^* and EtBr-treated *polg^−/−^*; (**e**) indicates significant difference between untreated *polg^+/−^* and EtBr-treated *polg^+/−^* animals. Significance achieved when *P* ≤ 0.05 by Mann–Whitney test.

Embryos were treated with EtBr for the first 6 dpf, after which they were rinsed and allowed to grow until mtDNA content and genotype were determined at 1, 2 or 3 wpf (corresponding to 1, 8 and 15 days after EtBr removal). Both WT and *polg^−/−^* animals exposed to EtBr had significantly lower mean dCt values at 1 week compared to unexposed fish of the same genotype (mean mtDNA copy numbers per cell of 77.8 versus 61.4 in WT and 31.2 versus 23 in *polg*^−/−^, a 21% and 26% reduction respectively), while values for treated and untreated *polg^+/−^* animals were not significantly different (Figure 6, Supplemental Table S3). At 2 wpf, all EtBr-treated fish had lower dCt values than untreated fish of the same genotype with reductions in mtDNA copy number per cell of 30% in WT, 20% in *polg*^+/−^ and 27% in *polg*^−/−^ animals compared to untreated animals of the same genotype. However, by 3 wpf both WT and *polg^+/−^* animals had dCt values that were indistinguishable from untreated fish (Figure 6, Supplemental Table S3), while EtBr-treated *polg^−/−^* animals remained significantly different from untreated fish of the same genotype with mtDNA copy number per cell of 20.2 compared to 27.8 in untreated *polg^−/−^* animals, a 27% decrease. These data show that 15 days after significant EtBr-induced mtDNA depletion, both *polg^+/−^* and WT animals are capable of repopulating their mtDNA to untreated levels. In contrast, EtBr treatment significantly reduced *polg^−/−^* mtDNA to a greater extent than *polg^−/−^* fish that were not treated, and treated animals were unable to induce mtDNA replication to repopulate their cells following depletion, with mtDNA content remaining depleted to the same level at all time points (Figure 6, Supplemental Table S3).

### The severity of the mtDNA depletion in *polg^−/−^* zebrafish is tissue-specific and correlates with respiration

**Figure 7.**
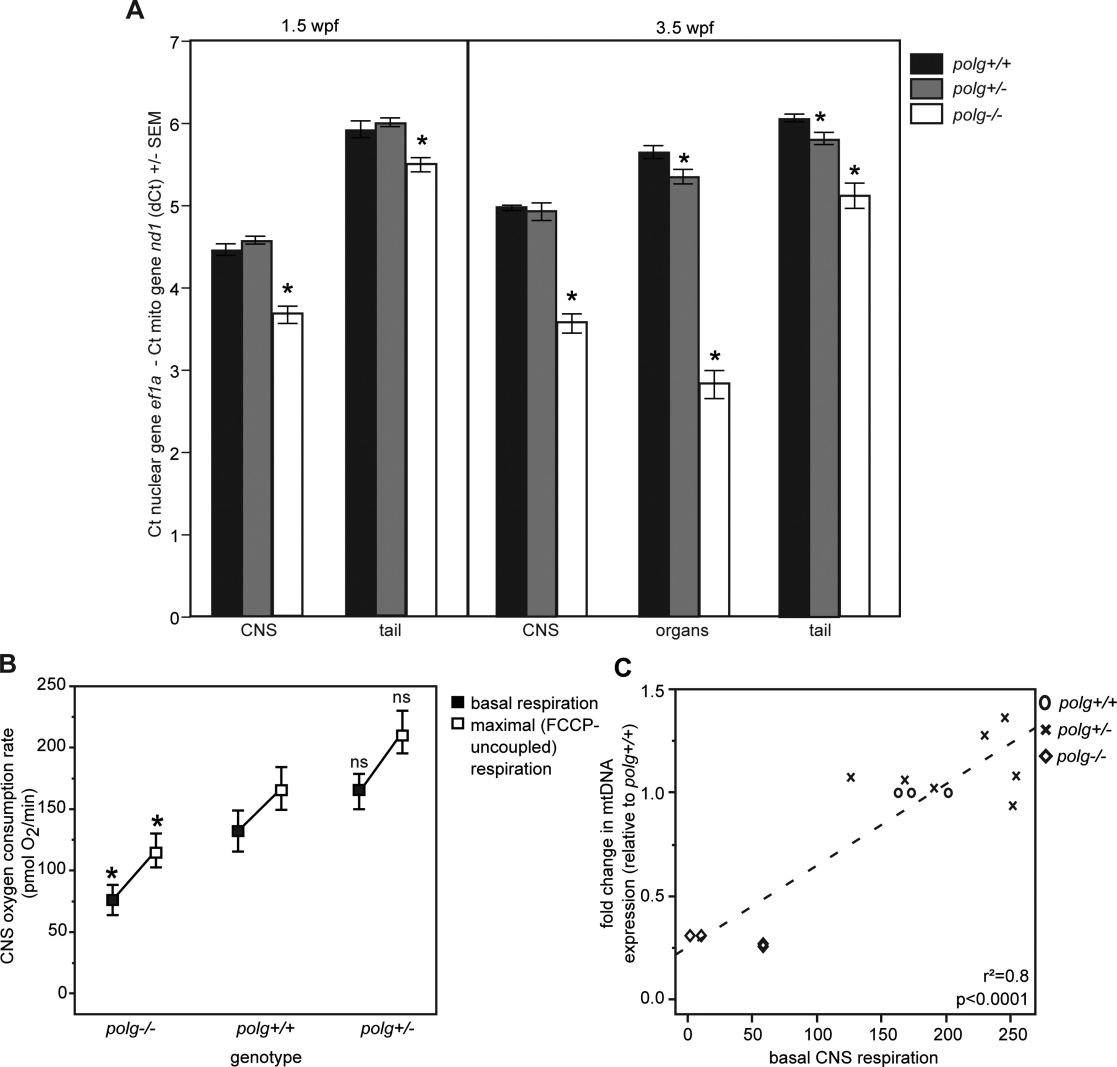
MtDNA content differs between tissues in *polg^−/−^* zebrafish, correlating with tissue specific levels of respiration. (**A**) Zebrafish tissue groups were isolated and analyzed for mtDNA content over time. Shown are mean dCt values with SEM *indicates significant difference from WT of the same tissue group; *P* ≤ 0.05 by Mann–Whitney test (see Supplemental Table S4 for fold change values, sample sizes and *P*-values). (**B**) Basal respiration (black) and maximal FCCP-uncoupled respiration (white) were measured from CNS tissue of 3 wpf zebrafish. *indicates significant difference from WT; *P* ≤ 0.05 ANOVA/Steel posthoc test (see Supplemental Table S5 for means, sample sizes and *P*-values). (**C**) There is a significant correlation between mtDNA content and respiration levels of CNS tissue. *polg^−/−^* samples (diamonds) cluster together with low mtDNA and respiration while *polg^+/−^* samples (crosses) display WT levels (circles) of mtDNA and respiration.

Patients with mitochondrial disease often have differences in the level of mtDNA depletion between tissues and even within the same tissue (44). This tissue heterogeneity can lead to misdiagnosis (46). We then measured mtDNA content was measured from three body areas in each animal: the CNS region composed of eyes and brain (‘CNS’); the tail region which includes somatic muscle and developing spinal cord (‘tail’); and the region containing the gills, heart and internal organs (‘organs’). At 1.5 wpf, the amount of DNA that could be recovered from the internal organs was insufficient for our analysis, thus only CNS and tail tissue groups were examined. At this time point, the relative abundance of mtDNA was significantly different between the tissues in all genotypes, where the amount of mtDNA in tail tissue was greater than that of CNS tissue. In WT animals, conversion of dCt to mean mtDNA copy number per cell (2^dCt^ × 2) yields a value of 43.8 mtDNA copies per cell in the CNS versus 119.4 in the tail, representing 2.7-fold more mtDNA present in the tail compared to the CNS. Similarly, in *polg*^+/−^ animals, the CNS contained a mean of 47.2 mtDNA copies per cell versus 126.2 in the tail, again revealing 2.7-fold more mtDNA in tail than CNS. In *polg*^−/−^ animals, tail tissue again had a greater number of mtDNA copies per cell than the CNS (90.6 for tail and 25.6 for CNS), however there was a much greater difference in these levels, where tail tissue had a 3.5-fold greater mtDNA copy number than CNS. (Figure 7A, Supplemental Table S4). MtDNA levels from heterozygous animal tissues did not differ from controls for either tissue group. However, *polg*^−/−^ CNS and tail tissues had significantly less mtDNA than WT (*P* = 0.05), with the CNS tissue more severely affected than tail (Figure 7A, Supplemental Table S4).

At 3.5 wpf, we were able to recover sufficient material to analyze all three tissue groups. Again, we noted tail tissue contained more mtDNA than organs, and organs had more mtDNA than CNS in WT and *polg^+/−^* animals. The mtDNA levels of WT and *polg^+/−^* CNS tissue were not significantly different (*P* = 0.93), but the organ and tail tissue of *polg^+/−^* animals contained slightly less mtDNA than WT tissues (*P* = 0.04 and 0.01, respectively, Figure 7A, Supplemental Table S4). While WT organ tissue contained an average of 99.8 mtDNA copies per cell, *polg*^+/−^ organ tissue only contained only 81, 18.8% less mtDNA than WT. Similarly, WT tail tissue contained 132.6 mtDNA copies per cell, whereas *polg*^+/−^ tail tissue measured only 111.4 copies, 16% less mtDNA than WT. MtDNA content from *polg^−/−^* animals was significantly less than WT animals across all three tissue groups (*P* < 0.01). Interestingly, WT and *polg*^+/−^ CNS tissue had lower dCt values, and thus mtDNA content, than organ tissue (see above). However, in *polg*^−/−^ animals the organ tissue measured the smallest dCt values of all groups (2.83 ± 0.17) corresponding to a mean mtDNA copy number per cell of just 14.2, indicating that this tissue was the most severely depleted of mtDNA overall. By comparison, the level of mtDNA depletion in tail tissue, while significant, was much less than that observed in organs or CNS (Figure 7A, Supplemental Table S4). MtDNA copy number per cell was 24.0 in the CNS and 69 in tail tissue of *polg*^−/−^ animals. This analysis indicates that, as also observed in humans, zebrafish show tissue-specific differences in mtDNA content when Polg is mutated.

Because low mtDNA content and Vdac1 protein levels in *polg^−/−^* animals indicates mitochondrial depletion at 3 wpf (Figure 3), we expected that *polg^−/−^* animals would also have impaired respiration. Using our previously developed methods to measure energetics from intact embryos (26), we did not detect respiratory defects in *polg* mutant embryos at 3 dpf, a time at which there is no mtDNA depletion (data not shown). As 3 wpf zebrafish are too large for whole animal measurements, we developed new methods to measure respiration from intact CNS tissue. With this approach, we found that *polg^+/−^* CNS basal respiration was not significantly different from WT (Figure 7B and Supplemental Table S5). However, *polg^−/−^* CNS tissue registered significantly lower basal respiration. Application of FCCP induces maximal respiration from mitochondria. Like basal respiration, FCCP-induced maximal respiration was not significantly different between WT and heterozygous mutant CNS, but the maximal respiration level of the *polg^−/−^* tissue was significantly lower than WT (*P* = 0.04). The difference between basal respiration and FCCP-uncoupled respiration is the ‘spare respiratory capacity,’ a measure of the ability of mitochondria to increase respiration to meet changing energy demands. Interestingly and importantly, we found that spare respiratory capacity was similar across all genotypes (Figure 7B and Supplemental Table S5). Upon completion of respiration measurements, tissue was collected and mtDNA levels were analyzed yielding a linear relationship between respiratory output and mtDNA levels (Figure 7C). Compared to WT samples, *polg^−/−^* CNS tissue had both low respiratory output and low mtDNA content, while *polg^+/−^* CNS samples clustered with WT samples (Figure 7C). Taken together, these data indicate that loss of mtDNA suffered by the *polg^−/−^* animals results in a concomitant and significant decrease in respiration, however as mitochondrial spare respiratory capacity was similar to WT, this indicates that mitochondrial functionality was not impacted.

## DISCUSSION

POLG is a major locus for human mitochondrial disease. However, understanding the progression of disease *in vivo* has been difficult, as mutations in POLG give rise to a wide range of complex diseases that greatly vary in tissue symptoms and age of onset, even with the same POLG genotype (4). With existing mouse models of Polg dysfunction, it is difficult to phenocopy the complexity of the human disease spectrum, as well as study the relationship between Polg function and early developmental processes. Thus, we have created a new model of mitochondrial disease, using TALEN gene editing technology to create stable *polg* mutations in zebrafish. Our zebrafish model is unique in that it enables us to examine the complex relationships between mtDNA content, energetics, growth and development in a whole organism over its lifespan.

### Heterozygous *polg* mutants are almost indistinguishable from WT zebrafish up to the juvenile stage

In humans, most of the 200+ pathogenic POLG mutations are autosomal recessive. Thus, the majority of individuals with heterozygous POLG mutations are thought to be asymptomatic for mitochondrial disease (4). We found that, despite having approximately 50% less WT Polg protein than controls (Figure 3B), *polg^+/−^* zebrafish had no sustained deficits compared to WT across many measurements, including survival rates (Figure 2), mtDNA levels (Figure 3A), lactate (glycolytic marker) levels (Figure 5B), and respiratory output (Figure 7). In addition, we found that *polg^+/−^* animals did not differ from WT in terms of growth and development (Figure 4) or rate of caudal fin regeneration after amputation (Figure 4D). We did observe a transient decrease in mtDNA content of 16% in *polg^+/−^* animals only at 3 wpf (Figure 3A) and a slight increase in ATP content (Figure 5A) at 1 wpf. However, these changes were not sustained to later time points. The slight decrease in total organismal mtDNA content at 3 wpf in whole animals is reflected in the observed decrease in mtDNA content in heterozygote organ (18.8% difference) and tail tissues (16% difference) compared to WT measured at 3.5 wpf (Figure 7A). Interestingly, CNS tissue mtDNA levels were not different between heterozygotes and WT, suggesting that the organ and tail tissues were more sensitive to the lower gene dose of WT Polg at this time point. However, this slight decrease in mtDNA content is likely not biologically relevant, as a 15% decrease in mtDNA found in heterozygous Polg mutant mice did not affect phenotype (14). Our calculated mtDNA copies per cell is in line with published work by Artuso et al. (35) who report approximately 100–150 mtDNA copies per cell in zebrafish aged 3 dpf to 8 dpf.

Given that *polg^+/−^* animals could maintain similar levels of mtDNA as WT, albeit with a slight decrease at 3 wpf, we wanted to test whether the single WT Polg allele was adequate to repopulate mtDNA after exposure to a mtDNA replication inhibitor. We adopted methods from cell culture to preferentially deplete mtDNA by treating fish with EtBr (47). By quantifying the time it takes for animals to replicate and replenish their mtDNA, this novel stress test provides an *in vivo* measure of mtDNA polymerase activity. *polg^+/−^* animals, like WT, had significant losses of mtDNA after exposure to EtBr (Figure 6). However, *polg^+/−^* fish were as capable of mtDNA replication and repopulation as their WT siblings, as both populations fully recovered mtDNA content by 3 wpf (Figure 6).

The observed similarity between *polg^+/−^* and WT zebrafish is in accordance with mouse studies demonstrating that heterozygous mice with the D257A Polg mutation were similar to WT with regard to development, longevity and mtDNA levels (14–16). However, Fuke *et al*. (48) recently showed that these same mice develop mtDNA deletions and eventually show neuromuscular defects. It is possible that abnormalities in *polg^+/−^* zebrafish may manifest later in life, and that susceptibilities to stressors such as EtBr may become apparent. It is possible that as these animals age, the differences we observe in whole animal and organ mtDNA content (Figures 3A and 7A) becomes more pronounced. This will be addressed in future studies in older-aged zebrafish.

### Why do homozygous *polg* zebrafish have severe mtDNA depletion early in life, yet survive until juvenile stage?

We were initially very surprised to recover any *polg^−/−^* zebrafish past embryogenesis, given that the mutation truncates the protein's critical polymerase domain (Figure 1) and homozygous mutant mice bearing a truncated Polg protein have close to complete loss of mtDNA and lose viability during embryogenesis (14). Yet, *polg^−/−^* zebrafish survive to 3 wpf before showing any significant increases in mortality (Figure 2). When we examined mtDNA content in *polg^−/−^* zebrafish at 3 dpf, there were no significant differences in mtDNA content compared to WT. However, just 3–4 days later (1 wpf) the *polg^−/−^* zebrafish revealed 48% lowered mtDNA content, which did not recover over their lifespan (Figure 3). Our *in vivo* mtDNA polymerase assay revealed that EtBr treatment further reduced mtDNA levels by 26% indicating that the mechanism for EtBr-induced mtDNA depletion occurs independent of mtDNA replication. Furthermore, unlike WT and *polg*^+/−^ animals, which fully recovered mtDNA content after EtBr was removed, *polg^−/−^* zebrafish were unable to recover mtDNA levels after EtBr removal, and levels remained 27% lower than untreated fish of the same age (Figure 6, Supplemental Table S2). This suggests that zebrafish harboring premature stop mutations in the active site of the Polg polymerase domain cannot adequately replicate mtDNA to WT levels. This assay does not attempt to assay mtDNA repair, another important function of Polg. It is possible that these *polg* mutations hinder the ability of Polg protein to perform repair under certain circumstances, an avenue that requires further study.

The initial survival of *polg^−/−^* animals may be due in part to the hypothesis that replication of mtDNA may not be necessary until later in development, as oocytes are pre-loaded with maternal mitochondria containing more than 10,000 times the amount of mtDNA/cell than is found in cells of adult zebrafish (35). In fact, expression of *polg* has been shown to be substantially lower in embryos and larvae up to 5 dpf, compared to adults (35), suggesting that ablating mtDNA replication in *polg*^−/−^ fish may not have substantial consequences until later in life.

Interestingly, there is a mismatch between the first indication of mtDNA depletion in *polg^−/−^* zebrafish (1 wpf) and the first evidence of developmental abnormalities (2–3 wpf; Figure 4). Unlike WT and *polg* heterozygous animals, *polg^−/−^* animals were smaller and disproportionate in their development, failing to reach the same developmental milestones as their WT and heterozygous siblings (Figure 4). *polg^−/−^* animals were also unable to regenerate their caudal fins after amputation (Figure 4D). Caudal fin regeneration occurs readily and rapidly in teleost fishes, relying on the expansion of a pluripotent progenitor cell population to repopulate the ablated tissue (49). Together, these data indicate that Polg protein and mitochondrial output are required for these complex developmental processes.

Looking more closely into the energetics of these fish, we were surprised to learn that *polg^−/−^* animals had similar levels of ATP as WT animals at 1 wpf (Figure 5). Furthermore, although *polg^−/−^* animals begin to die by 3 wpf (Figure 2), we also saw no significant differences in ATP levels between WT, *polg*^+/−^ or *polg^−/−^* animals at this time (Figure 5A). Glycolysis can be used to generate ATP without mitochondrial involvement; it is a major energy source for dividing progenitor cells (50) and can be induced in situations when OXPHOS is impaired (51–53). Though we hypothesized that *polg^−/−^* zebrafish employ glycolysis to compensate for a lack of OXPHOS-generated ATP that is expected when Polg is mutated, we found no significant differences in lactate content (Figure 5B), suggesting that glycolysis was not more active in *polg*^−/−^ animals.

MtDNA content of *polg^−/−^* zebrafish remained substantially depleted until time of death, but it is important to point out that it was not zero; the lowest mean mtDNA copy number per cell was 23.2 at 3 wpf, compared to 96 in WT fish of the same age. This small mtDNA population could still be actively transcribed and the resulting mRNA translated, and thereby could contribute to mitochondrial ATP production. As *polg^−/−^* animals had similar ATP content to WT, we measured respiration from the CNS of these animals to assess mitochondrial energetics. We found that *polg^−/−^* zebrafish had significantly decreased basal and maximal respiration compared to WT or *polg^+/−^* zebrafish (Figure 7B), and that levels of respiration correlated with mtDNA content (Figure 7C). This analysis showed again that *polg^+/−^* and WT animals clustered together, but that *polg^−/−^* animals grouped separately, with low respiration and low mtDNA levels (Figure 7C). This is not unexpected, given that protein levels of a major component of the OXPHOS machinery, CoxIV, were also lower in these animals (data not shown). We tested the ability of this tissue (and the mitochondria within) to respond to the uncoupling compound FCCP, which provides a measure of the spare respiratory capacity when basal respiration is subtracted (54). Importantly, this experiment showed that mitochondria from *polg^−/−^* animals had similar spare respiratory capacity to WT, indicating that mitochondria within these fish could function normally. When we examined mtDNA from all genotypes, we did not observe a difference in the presence of large mtDNA deletions among these fish (Supplemental Figure S4); an observation which supports the hypothesis that mtDNA remaining in *polg^−/^*^−^ fish are able to contribute production of OXPHOS complexes. Since basal respiration of *polg^−/−^* zebrafish was inhibited (Figure 7), but total body ATP and lactate levels were not different to WT (Figure 5), it is our hypothesis that these fish are responding to the decreased mtDNA levels (and by extension reduced respiratory output) by slowing cell division resulting in the observed developmental and growth abnormalities. It has been demonstrated that fish can suppress cell growth and proliferation in order to conserve ATP under hypoxic conditions (55), and goby fish have been shown to increase expression of genes known to suppress cell growth and proliferation under similar stress conditions (56). Our ATP analysis only measures total body ATP content; if these animals are producing less ATP and are using less ATP, this would result in no net change in ATP content as observed. Importantly, the mitochondria in *polg^−/^*^−^ fish maintained similar levels of spare respiratory capacity as WT, suggesting normal mitochondrial function. Additionally, the fact that total ATP content did not differ significantly in these animals suggests that while the mitochondria were operating at lower basal levels than WT animals, the slower cell division and growth resulted in less ATP being used. This may result in a situation where a switch to glycolysis is not needed as supported by our observation that lactate levels in *polg*^−/−^ animals were not significantly different from WT.

We also showed that the degree of mtDNA depletion differs between tissues, and that the amount of mtDNA correlates with respiration of that tissue (Figure 7C). In *polg^−/−^* zebrafish at 1.5 wpf, there is less mtDNA in both CNS and tail tissue compared to WT animals (Figure 7A). Furthermore, for *polg^−/−^* zebrafish the CNS tissue is more severely depleted of mtDNA than tail tissue (Figure 7A). This finding recapitulates what is observed in many human patients with POLG disease mutations, where CNS is a high energy tissue that is generally the first to show symptoms in mitochondrial disease (57). For *polg^−/−^* zebrafish near the end of their lives at 3.5 wpf, CNS, organ and tail tissues all exhibited significantly lower mtDNA than WT tissues. Interestingly, while mtDNA content in WT and *polg*^+/−^ zebrafish was lowest in CNS, higher in organs, and highest in tails, in *polg^−/−^* animals the organ tissue was the most depleted tissue, registering the lowest amount of mtDNA among all groups with a mean mtDNA copy number per cell of 14.2 compared to 99.8 in WT organs (Figure 7A). CNS tissue was the next most affected with a mean mtDNA copy number per cell of 24 compared to 62.6 in WT, while tail tissue had more normal but still significantly depleted levels of mtDNA (mean mtDNA copy number per cell of 69 compared to 132.6 in WT). The fact that organ and CNS tissues are most affected in homozygous animals is compelling, given that liver dysfunction, along with intractable seizures, is the hallmark of the liver-CNS disease known as Alpers syndrome, the main childhood mitochondrial disease caused by POLG mutations (58). Further studies will be conducted to determine the extent to which loss of mtDNA in these tissues produce organ failure and death, such as is seen in humans. Most importantly, these data indicate that maintaining proper mtDNA homeostasis is more difficult in certain tissues when Polg polymerase function is impacted.

In conclusion, this zebrafish model of mitochondrial disease provides unique opportunities for studying Polg dysfunction from multiple angles. Little is known about the susceptibility of individuals with POLG mutations to stressors that include environmental contaminants, drug exposures and dietary deficiencies, which could address the variability in mitochondrial disease phenotypes in people with the same POLG mutation. With this molecular and developmental framework, we are now able to quantify the changes induced by toxins or treatments, a clear niche that a zebrafish model can easily address. Furthermore, in contrast to mice, *polg* homozygous mutant zebrafish live through early embryonic development and survive weeks after their mtDNA is severely depleted. The fact that these fish survive for as long as they do provides a unique opportunity to study why certain tissue types are preferentially affected by loss of mtDNA, and the conditions that can promote different disease outcomes. This knowledge will be important for developing effective treatment strategies for this currently incurable set of diseases.

## Supplementary Material

SUPPLEMENTARY DATA
