# Supplementary material for: Zebrafish lacking functional DNA polymerase gamma survive to juvenile stage, despite rapid and sustained mitochondrial DNA depletion, altered energetics and growth
Source: Nucleic Acids Res. 2015 Oct 30;43(21):10338–52. doi: 10.1093/nar/gkv1139 (PMC4666367; doi:10.1093/nar/gkv1139)
Supplement: SUPPLEMENTARY DATA [file supp_43_21_10338__index.html]

Zebrafish lacking functional DNA polymerase gamma survive to juvenile stage, despite rapid and sustained mitochondrial DNA depletion, altered energetics and growth — SUPPLEMENTARY DATA 

# Zebrafish lacking functional DNA polymerase gamma survive to juvenile stage, despite rapid and sustained mitochondrial DNA depletion, altered energetics and growth

## SUPPLEMENTARY DATA

- SUPPLEMENTARY DATA
