# Supplementary material for: Zebrafish lacking functional DNA polymerase gamma survive to juvenile stage, despite rapid and sustained mitochondrial DNA depletion, altered energetics and growth
Source: Nucleic Acids Res. 2015 Oct 30;43(21):10338–52. doi: 10.1093/nar/gkv1139 (PMC4666367; doi:10.1093/nar/gkv1139)
Supplement: SUPPLEMENTARY DATA [file supp_gkv1139_nar-02607-h-2015-File009.pdf]

Supplemental Table 1, mean dCt values (Ct of nuclear gene *ef1a* – Ct of mitochondrial gene *nd1*) of mtDNA content analysis with SEM and sample size. Shown are all individual genotypes analyzed. Final two rows are combined genotypes plotted in Figure 3. p-values calculated with Mann-Whitney nonparametric test compared to *polg*$^{+/+}$ of same age. Bold lettering indicates significant difference (p≤0.05).

genotype	0.5 wpf	1 wpf	1.5 wpf	2 wpf	2.5 wpf	3 wpf	4 wpf
polg$^{+/+}$	5.65 ± 0.08<br>n = 24<br>p = N/A	5.12 ± 0.16<br>n = 17<br>p = N/A	5.05 ± 0.21<br>n = 14<br>p = N/A	4.95 ± 0.08<br>n = 24<br>p = N/A	5.86 ± 0.21<br>n = 8<br>p = N/A	5.59 ± 0.1<br>n = 16<br>p = N/A	6.14 ± 0.13<br>n = 13<br>p = N/A
polg$^{+/muz119}$	5.74 ± 0.06<br>n = 38<br>p = 0.25	5.43 ± 0.11<br>n = 24<br>p = 0.61	5.29 ± 0.12<br>n = 22<br>p = 0.16	4.83 ± 0.07<br>n = 17<br>p = 0.11	6.10 ± 0.11<br>n = 17<br>p = 0.31	5.35 ± 0.1<br>n = 7<br>p = 0.12	5.86 ± 0.12<br>n = 19<br>p = 0.23
polg$^{+/muz120}$	5.54 ± 0.13<br>n = 10<br>p = 0.55	5.23 ± 0.04<br>n = 5<br>p = 0.39	4.95 ± 0.27<br>n = 10<br>p = 0.58	5.10 ± 0.05<br>n = 19<br>p = 0.36	6.46 ± 0.08<br>n = 5<br>p = 0.14	5.34 ± 0.09<br>n = 10<br>p = 0.07	5.65 ± 0.28<br>n = 2<br>p = 0.20
polg$^{+/muz121}$	5.74 ± 0.1<br>n = 21<br>p = 0.35	5.44 ± 0.16<br>n = 18<br>p = 0.20	5.38 ± 0.2<br>n = 12<br>p = 0.29	4.86 ± 0.14<br>n = 10<br>p = 0.36	6.07 ± 0.17<br>n = 3<br>p = 0.36	5.74 ± 0.09<br>n = 17<br>p = 0.41	5.97 ± 0.18<br>n = 9<br>p = 0.53
polg$^{muz119/muz119}$	**5.19 ± 0.19<br>n = 5<br>p = 0.04**	**3.58 ± 0.06<br>n = 3<br>p = 0.02**	4.86 ± 0.07<br>n = 4<br>p = 0.31	**3.25 ± 0.18<br>n = 2<br>p = 0.02**	**4.28 ± 0.55<br>n = 4<br>p = 0.01**	**3.96 ± 0.17<br>n = 2<br>p = 0.029**	**4.75<br>n = 1**
polg$^{muz119/muz120}$	5.44 ± 0.27<br>n = 5<br>p = 0.44	**4.52 ± 0.13<br>n = 5<br>p = 0.006**	**3.68 ± 0.21<br>n = 3<br>p = 0.03**	**3.95 ± 0.10<br>n = 6<br>p = 0.0007**	**4.04 ± 0.48<br>n = 4<br>p = 0.009**	**3.41 ± 0.17<br>n = 7<br>p =0.0002**	
polg$^{muz120/muz120}$				**3.39 ± 0.10<br>n = 4<br>p = 0.002**			
polg$^{muz121/muz121}$						**3.79 ± 0.17<br>n = 5<br>p = 0.001**	
polg$^{muz119/muz121}$	5.56 ± 0.1<br>n = 14<br>p = 0.41	4.7 ± 0.55<br>n = 2<br>p = 0.84	4.59 ± 0.21<br>n= 6<br>p = 0.11		**4.00 ± 0.05<br>n = 8<br>p = 0.0009**	**3.25 ± 0.22<br>n = 2<br>p = 0.03**	

polg$^{muz120/muz121}$	**4.40 ± 0.1**<br>**n = 7**<br>**p = 0.0006**	**3.75 ± 0.14**<br>**n = 9**<br>**p = 0.0003**	**3.87 ± 0.2**<br>**n = 6**<br>**p = 0.01**	**3.15 ± 0.11**<br>**n = 6**<br>**p = 0.0002**	**4.72 ± 0.07**<br>**n = 2**<br>**p = 0.05**		
polg$^{+/muz191}$,<br>*polg*$^{+/muz120}$	5.70 ± 0.05<br>n = 48<br>p = 0.62	5.40± 0.1<br>n = 29<br>p = 0.48	5.19 ± 0.12<br>n = 32<br>p = 0.42	4.98 ± 0.05<br>n = 36<br>p = 0.74	6.18 ± 0.09<br>n = 22<br>p = 0.19	**5.34 ± 0.08**<br>**n = 17**<br>**p = 0.04**	5.84 ± 0.52<br>n = 21<br>p = 0.17
polg$^{muz119/muz119}$,<br>*polg*$^{muz119/muz120}$,<br>*polg*$^{muz120/muz120}$	5.32 ± 0.16<br>n = 10<br>p = 0.06	**4.17 ± 0.19**<br>**n = 8**<br>**p = <0.001**	**4.35 ± 0.25**<br>**n = 7**<br>**p = 0.04**	**3.65 ± 0.11**<br>**n = 12**<br>**p = <0.001**	**4.16 ± 0.34**<br>**n = 8**<br>**p = <0.001**	**3.53 ± 0.15**<br>**n = 9**<br>**p = <0.001**	

Supplemental Table 2, mean growth and development metrics measured at three time points. Shown are mean values with SEM and sample size as well as p-values calculated with Mann-Whitney nonparametric test to WT of the same age. Data from this table appears in Figure 4.

		WT	*polg*$^{+/-}$	*polg*$^{-/-}$
larval length (mm)	1 wpf	3.8 ± 0.02 (n = 39)	3.7 ± 0.03 (n = 46, p = 0.84)	3.8 ± 0.03 (n = 20, p = 0.15)
	2 wpf	4.6 ± 0.11 (n = 16)	4.5 ± 0.09 (n = 32, p = 0.67)	4.1 ± 0.06 (n = 21, p = 0.36)
	3 wpf	4.8 ± 0.06 (n = 60)	5.0 ± 0.15 (n = 37, p = 0.96)	**4.5 ± 0.05 (n = 37, p = 0.0002)**
body width (µm)	1 wpf	249.1 ± 4.21 (n = 34)	250.8 ± 3.51 (n = 42, p = 0.98)	355.3 ± 3.89 (n = 17, p = 0.64)
	2 wpf	303.3 ± 11.82 (n = 20)	322.0 ± 10.53 (n = 37, p = 0.48)	**260.1 ± 8.32 (n = 22, p = 0.02)**
	3 wpf	340.1 ± 10.1 (n = 50)	406.8 ± 31.8 (n = 31, p = 0.64)	**280.6 ± 7.6 (n = 30, p = 0.0002)**
eye diameter (µm)	1 wpf	347.6 ± 2.2 (n = 63)	343.2 ± 3.0 (n = 53, p = 0.26)	**333.3 ± 3.22 (n = 35, p = 0.0004)**
	2 wpf	410.6 ± 12.02 (n = 21)	430.4 ± 9.86 (n = 33, p = 0.41)	**363.5 ± 6.86 (n = 23, p = 0.01)**
	3 wpf	442.1 ± 7.18 (n = 60)	468.4 ± 14.79 (n = 45, p = 0.83)	**396.7 ± 16.1 (n = 18, p = <0.0001)**
caudal fin regeneration (µm)	1 wpf	33.2 ± 3.34 (n = 27)	33.1 ± 3.02 (n = 29, p = 0.95)	23.2 ± 3.98 (n = 13, p = 0.17)
	2 wpf	61.1 ± 10.27 (n = 10)	85.1 ± 9.8 (n = 21, p = 0.19)	**27.0 ± 3.5 (n = 8, p = 0.01)**
	3 wpf	72.6 ± 9.28 (n = 39)	88.9 ± 15.65 (n = 30, p = 0.96)	**23.5 ± 2.86 (n = 29, p = <0.0001)**

Supplemental Table 3A, mean dCt values (Ct of nuclear gene *ef1a* – Ct of mitochondrial gene *nd1*) of EtBr stress test with SEM and sample size. Data are plotted in Figure 6.

genotype and treatment group	1 wpf	2 wpf	3 wpf
polg$^{+/+}$ **untreated**	5.28 ± 0.09<br>n = 18	5.54 ± 0.07<br>n = 14	5.60 ± 0.07<br>n = 17
polg$^{+/+}$ **EtBr**	4.94 ± 0.09<br>n = 18	5.02 ± 0.08<br>n = 14	5.44 ± 0.08<br>n = 20
polg$^{+/-}$ **untreated**	5.01 ± 0.07<br>n = 22	5.16 ± 0.07<br>n = 22	5.53 ± 0.06<br>n = 20
polg$^{+/-}$ **EtBr**	4.91 ± 0.05<br>n = 21	4.84 ± 0.07<br>n = 20	5.33 ± 0.07<br>n = 20
polg$^{-/-}$ **untreated**	3.96 ± 0.12<br>n = 18	4.01 ± 0.12<br>n = 19	3.80 ± 0.12<br>n= 10
polg$^{-/-}$ **EtBr**	3.52 ± 0.10<br>n = 17	3.56 ± 0.1<br>n = 21	3.34 ± 0.10<br>n = 17

Supplemental Table 3B, p-values by nonparametric Mann-Whitney tests for EtBr stress test dCt values. Bold lettering indicates significant difference (p≤0.05).

**wpf**	**genotype and treatment**	vs *polg*$^{+/+}$ untreated	vs *polg*$^{+/+}$ EtBr	vs *polg*$^{+/-}$ untreated	vs *polg*$^{+/-}$ EtBr	vs *polg*$^{-/-}$ untreated	vs *polg*$^{-/-}$ EtBr
1	*polg*$^{+/+}$ untreated	--	**0.008**	**0.02**	**0.001**	**<0.0001**	**<0.0001**
1	*polg*$^{+/+}$ EtBr		--	0.47	0.54	**<0.0001**	**<0.0001**
1	*polg*$^{+/-}$ untreated			--	0.11	**<0.0001**	**<0.0001**
1	*polg*$^{+/-}$ EtBr				--	**<0.0001**	**<0.0001**
1	*polg*$^{-/-}$ untreated					--	**0.012**
1	*polg*$^{-/-}$ EtBr						--
2	*polg*$^{+/+}$ untreated	--	**0.0005**	**0.004**	**0.0005**	**<0.0001**	**<0.0001**
2	*polg*$^{+/+}$ EtBr		--	0.25	0.10	**<0.0001**	**<0.0001**
2	*polg*$^{+/-}$ untreated			--	**0.006**	**<0.0001**	**<0.0001**
2	*polg*$^{+/-}$ EtBr				--	**<0.0001**	**<0.0001**
2	*polg*$^{-/-}$ untreated					--	**0.005**
2	*polg*$^{-/-}$ EtBr						--
3	*polg*$^{+/+}$ untreated	--	0.18	0.37	**0.013**	**<0.0001**	**<0.0001**
3	*polg*$^{+/+}$ EtBr		--	0.66	0.20	**<0.0001**	**<0.0001**
3	*polg*$^{+/-}$ untreated			--	0.06	**<0.0001**	**<0.0001**
3	*polg*$^{+/-}$ EtBr				--	**<0.0001**	**<0.0001**
3	*polg*$^{-/-}$ untreated					--	**0.005**
3	*polg*$^{-/-}$ EtBr						--

Supplemental Table 4A, mean dCt values (Ct of nuclear gene *ef1a* – Ct of mitochondrial gene *nd1*) of tissue analysis with SEM and sample size. Data are plotted in Figure 7A.

genotype	part	1.5 wpf	3.5 wpf
polg$^{+/+}$	CNS	4.45 ± 0.07<br>n = 4 (pools of 3)	4.97 ± 0.03<br>n = 9 individuals
	organs	N/A	5.64 ± 0.08<br>n = 9 individuals
	tail	5.90 ± 0.10<br>n = 4 (pools of 3)	6.05 ± 0.05<br>n = 9 individuals
polg$^{+/-}$	CNS	4.56 ± 0.05<br>n = 4 (pools of 3)	4.93 ± 0.11<br>n = 9 individuals
	organs	N/A	5.34 ± 0.09<br>n = 9 individuals
	tail	5.98 ± 0.05<br>n = 4 (pools of 3)	5.80 ± 0.07<br>n = 9 individuals
polg$^{-/-}$	CNS	3.68 ± 0.10<br>n = 3 (pools of 3)	3.58 ± 0.12<br>n = 8 individuals
	organs	N/A	2.83 ± 0.17<br>n = 8 individuals
	tail	5.50 ± 0.09<br>n = 3 (pools of 3)	5.11 ± 0.15<br>n = 8 individuals

Supplemental Table 4B, p-values by nonparametric Mann-Whitney tests for tissue parts analysis at 1.5 wpf. Bold lettering indicates significant difference (p≤0.05).

	polg$^{+/+}$ **CNS**	***polg***$^{+/+}$ **tail**	***polg***$^{+/-}$ **CNS**	***polg***$^{+/-}$ **tail**	***polg-/-*** **CNS**	***polg***$^{-/-}$ **tail**
polg$^{+/+}$ **CNS**	--	**0.03**	0.31	**0.03**	**0.05**	**0.05**
polg$^{+/+}$ **tail**		--	**0.03**	0.66	**0.05**	**0.05**
polg$^{+/-}$ **CNS**			--	**0.03**	**0.05**	**0.05**
polg$^{+/-}$ **tail**				--	**0.05**	**0.05**
polg$^{-/-}$ **CNS**					--	0.08
polg$^{-/-}$ **tail**						--

Supplemental Table 4C, p-values by nonparametric Mann-Whitney tests for tissue parts analysis at 3.5 wpf. Bold lettering indicates significant difference (p≤0.05).

	polg$^{+/+}$ CNS	*polg*$^{+/+}$ organs	*polg*$^{+/+}$ tail	*polg*$^{+/-}$ CNS	*polg*$^{+/-}$ organs	*polg*$^{+/-}$ tail	*polg*$^{-/-}$ CNS	*polg*$^{-/-}$ organs	*polg*$^{-/-}$ tail
polg$^{+/+}$ CNS	--	**0.0004**	**0.0004**	0.93	**0.01**	**0.0004**	**0.0006**	**0.0006**	0.23
polg$^{+/+}$ organs		--	**0.002**	**0.0006**	**0.04**	0.16	**0.0006**	**0.0006**	**0.01**
polg$^{+/+}$ tail			--	**0.0004**	**0.0004**	**0.01**	**0.0006**	**0.0006**	**0.0006**
polg$^{+/-}$ CNS				--	**0.03**	**0.0004**	**0.0006**	**0.0006**	0.23
polg$^{+/-}$ organs					--	**0.003**	**0.0006**	**0.0006**	0.36
polg$^{+/-}$ tail						--	**0.0006**	**0.0006**	**0.003**
polg$^{-/-}$ CNS							--	**0.005**	**0.0009**
polg$^{-/-}$ organs								--	**0.0009**
polg$^{-/-}$ tail									--

Supplemental Table 5A, Mean respiration rates with ANOVA and Steel post hoc tests for significance. Data is plotted in Figure 7B.

measurement	genotype	N	mean (pmol O_2/min)	ANOVA F-value	Steel post hoc with control p-value
basal respiration	*polg*$^{+/+}$	17	132.2 ± 16.7	0.0002	-
	polg$^{+/-}$	28	164.4 ± 14.4		0.25
	polg$^{-/-}$	21	76.1 ± 12.3		0.03
maximal respiration (FCCP-uncoupled respiration)	*polg*$^{+/+}$	17	166.8 ± 17.4	0.28	-
	polg$^{+/-}$	28	212.8 ± 17.3		-
	polg$^{-/-}$	21	116.4 ± 13.8		-
spare capacity (FCCP-basal respiration)	*polg*$^{+/+}$	17	34.6 ± 4.6	0.0004	-
	polg$^{+/-}$	28	48.4 ± 6.7		0.14
	polg$^{-/-}$	21	40.3 ± 5.4		0.04

Supplemental Table 5B, Respiration rates and mtDNA levels. Data is plotted in Figure 7C.

fish ID	genotype	basal respiration (pmol O_2/min)	mtDNA content (relative to *+/+*)
na	*polg*$^{+/+}$	173.23	-
na	*polg*$^{+/+}$	201.72	-
na	*polg*$^{+/+}$	163.03	-
1	*polg*$^{-/-}$	58.43	0.27
2	*polg*$^{-/-}$	58.43	0.27
3	*polg*$^{-/-}$	1.68	0.31
4	*polg*$^{-/-}$	10.47	0.31
5	*polg*$^{+/-}$	251.93	0.94
6	*polg*$^{+/-}$	230.25	1.28
7	*polg*$^{+/-}$	254.36	1.08
8	*polg*$^{+/-}$	168.54	1.07
9	*polg*$^{+/-}$	245.71	1.36
10	*polg*$^{+/-}$	126.47	1.08
11	*polg*$^{+/-}$	190.89	1.02

SUPPLEMENTAL FIGURE LEGENDS

Supplemental Fig. 1. TALEN vectors used to construct *polg* mutant zebrafish. (A) Vector diagrams (B) Sequence for targeting region of each vector (C) Schematic representation of human POLG and zebrafish Polg proteins.

Supplemental Fig. 2. High-resolution melt (HRM) analysis allows distinction between genotypes. Shown are the HRM curves obtained for sequence verified samples of each genotype (red) overlaid with WT curves (black). Each genotype yielded a unique curve which can be easily distinguished with the exception of $polg^{muz120/muz120}$ whose curve is very similar to that of WT zebrafish.

Supplemental Fig. 3. mtDNA content analysis for all genotypes assayed. dCt values (Ct *nd1* – Ct *ef1a*) are plotted over time for all genotypes. Some genotypes are only represented by one time point. See also Supplemental Table 1.

Supplemental Fig. 4. Deletion analysis. Long-range PCR was performed using Invitrogen Platinum *Taq* High Fidelity DNA polymerase in 25 µL reactions with 1 µL $MgSO_4$, 0.4 µM dNTPs (Invitrogen), 0.2 µM each primer (F-CAAACCCCCTTACCCCCTTAC; R- AGGGGTCTAACCTCCGATCT), 8.5 ng genomic DNA isolated from individual larvae, and 0.1 µL polymerase. Cycling conditions were 94˚C for 30 s followed by 38 cycles of 94˚C for 30 s, 60˚C for 30 s, 68˚C for 16 min followed by a final extension of 68˚C for 10 min. Reactions were subjected to agarose gel electrophoresis and gels imaged using GBOX (Syngene). Full length amplification with these primers results in 16 kb band. (A) Plotted is the mean amplicon size (bars) with the amplicon length of each sample overlaid as X. The number of larvae at each time point is shown above each bar. Data is grouped by genotype. (B) Representative gel of mtDNA deletions from individual fish.

Supplemental Fig. 5. Growth characteristics for all genotypes assayed. Shown are body length, body width, eye diameter, and caudal fin regrowth metrics for all genotypes assayed. Sample size, mean and SEM are shown to the right of each graph.

Supplemental Figure 1

A.

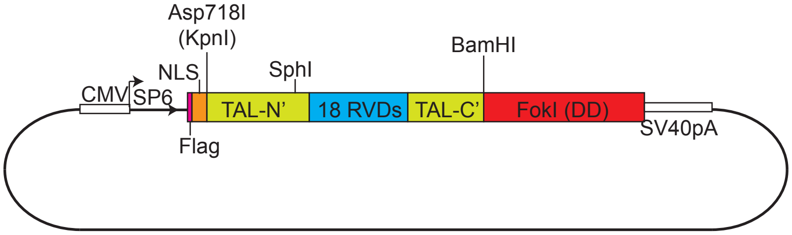

pCS2TAL3DD-backbone: 5,768bp
18 RVDs: 1836
Total: 7604bp

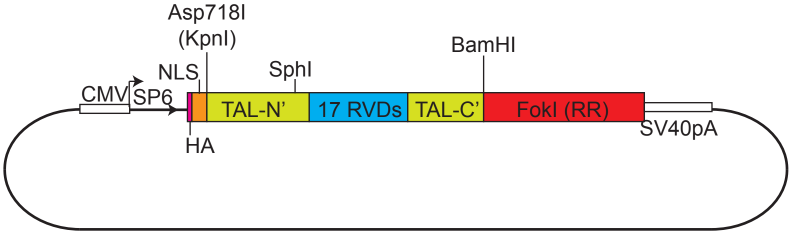

pCS2TAL3RR-backbone: 5,768bp
17 RVDs: 1734
Total: 7502bp

B.

Target Sequence (+ strand; 5' to 3')

T GCCAAGGTCTTCAACTAT gggcgcatttatggagc AGGTCAGCCATTTGCAG A

TALEN name	length (bp binding DNA sequence)	RVD sequence
polg-TALEN-L	18 bp T GCCAAGGTCTTCAACTAT	NN HD HD NI NI NN NN NG HD NG NG HD NI NI HD NG NI NG
polg-TALEN-R	17 bp T CTGCAAATGGCTGACCT	HD NG NN HD NI NI NI NG NN NN HD NG NN NI HD HD NG
spacer	17 bp gggcgcatttatggagc	

C.

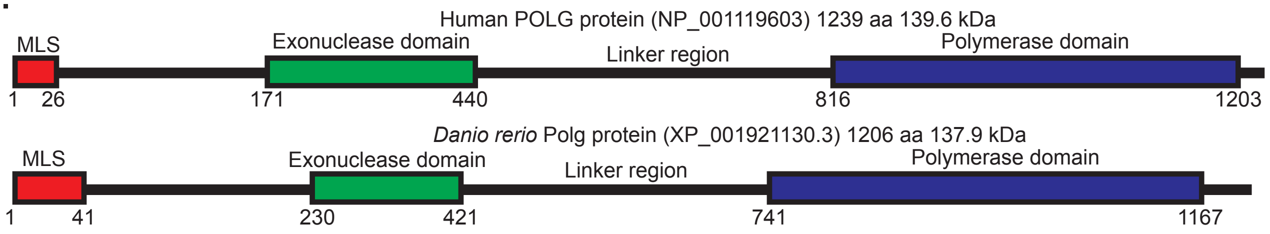

Supplemental Figure 2

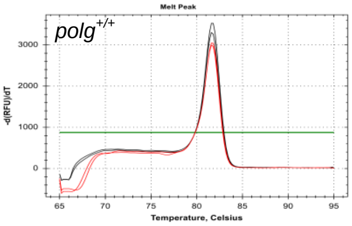

Heterozygous mutant genotypes

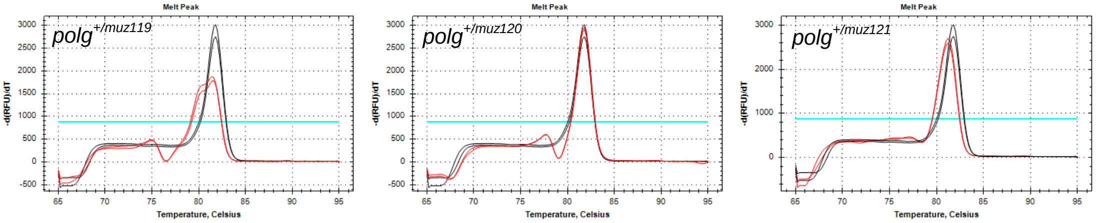

Homozygous mutant genotypes

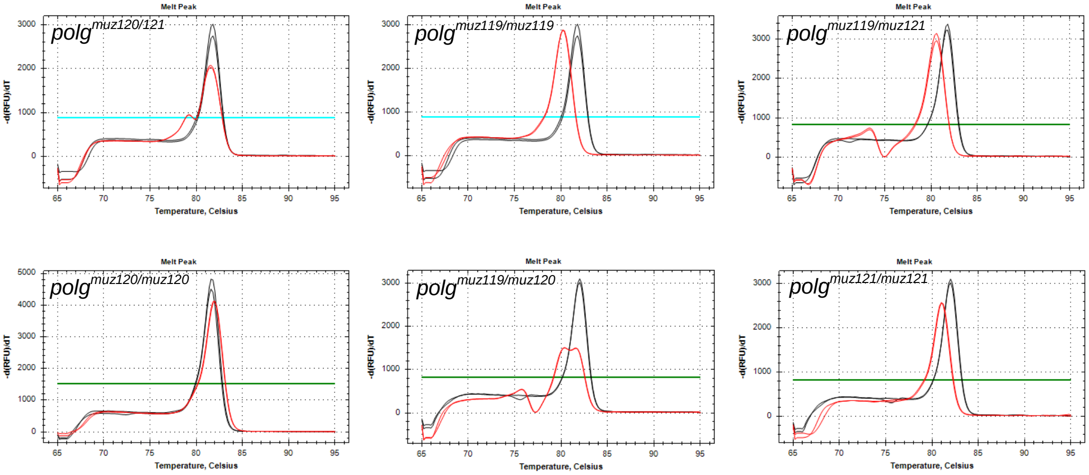

Supplemental Figure 3

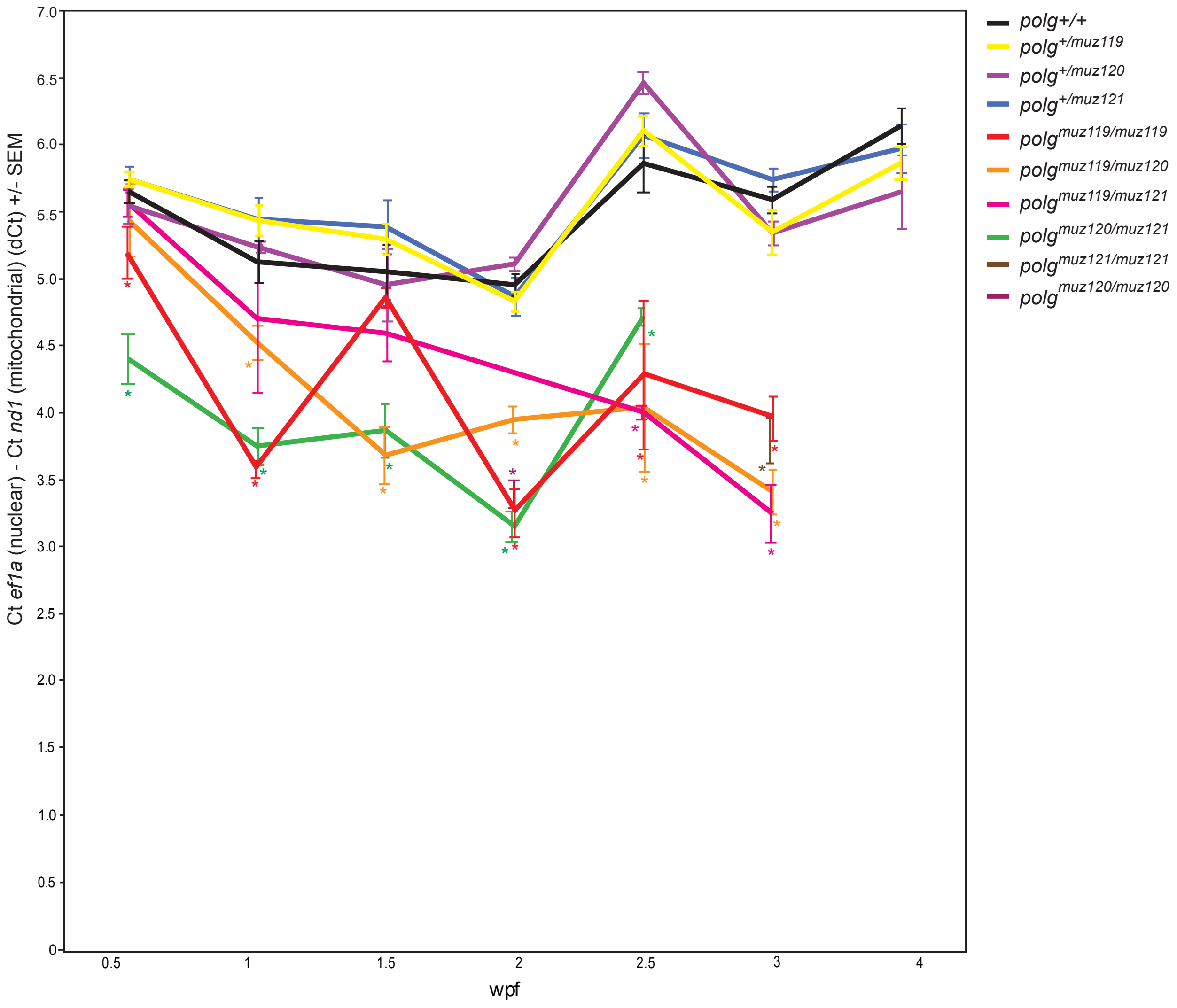

Supplementary Figure 4

A.

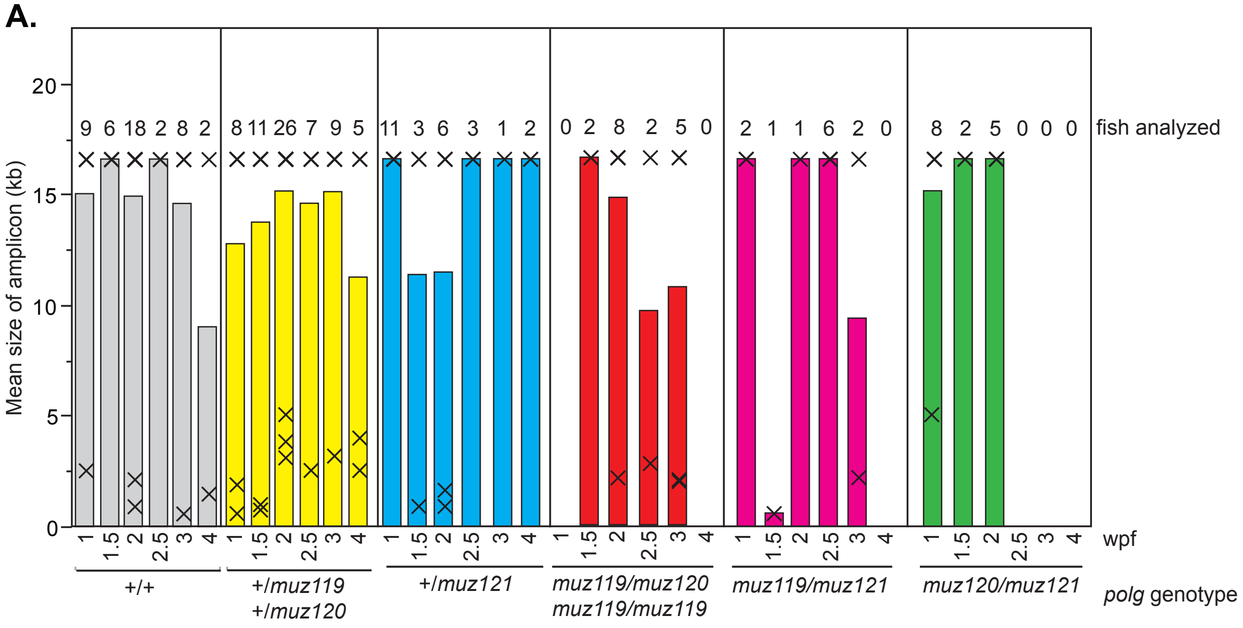

B.

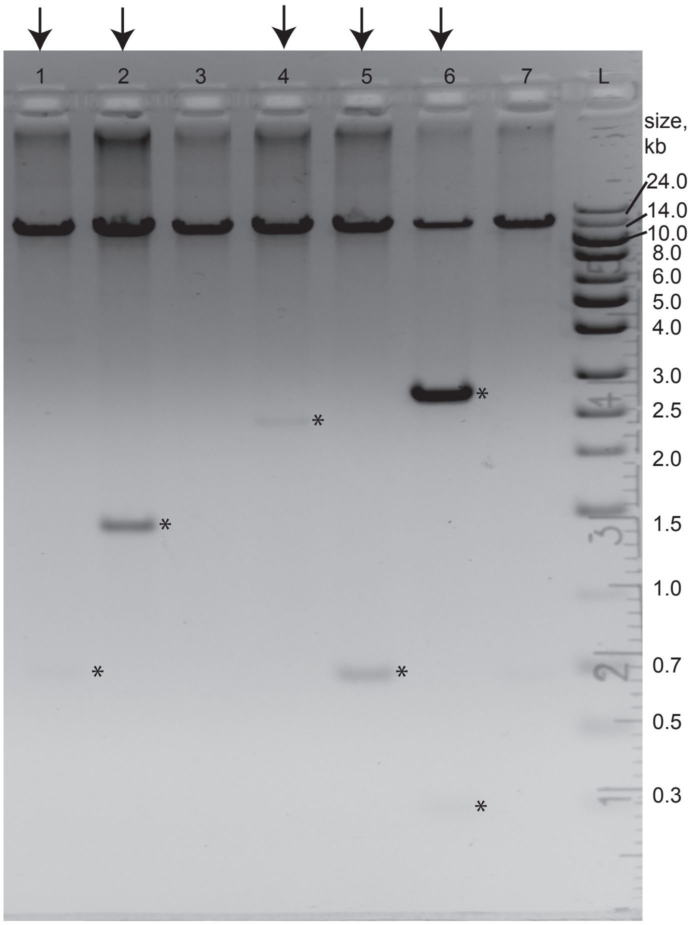

1. 4 wpf +/*muz119*
2. 4 wpf +/+
3. 4 wpf +/*muz120*
4. 4 wpf +/*muz120*
5. 1.5 wpf +/*muz119*
6. 2.5 wpf *muz119/muz120*
7. 2.5 wpf *muz119/muz120*

L. Ladder

Supplemental Figure 5

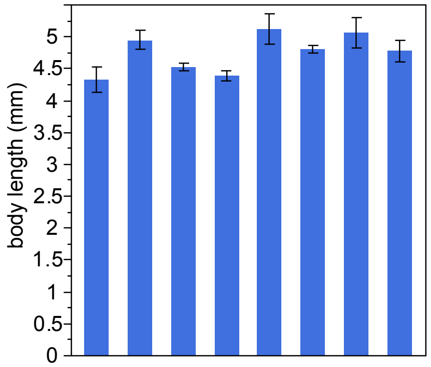

genotype	N	mean	SEM
polg$^{muz120/121}$	3	4.33333	0.20276
polg$^{muz119/121}$	2	4.95000	0.15000
polg$^{muz119/119}$	18	4.53111	0.06051
polg$^{muz119/120}$	19	4.39368	0.08303
polg$^{+/119}$	20	5.12350	0.23884
polg$^{+/+}$	60	4.80933	0.05989
polg$^{+/121}$	21	5.07143	0.23956
polg$^{+/120}$	17	4.78176	0.16665

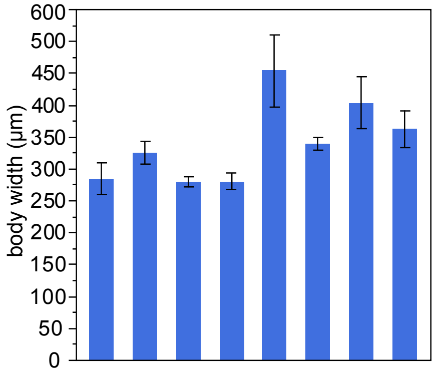

genotype	N	mean	SEM
polg$^{muz120/121}$	3	284.627	25.092
polg$^{muz119/121}$	2	326.177	18.162
polg$^{muz119/119}$	15	279.817	8.424
polg$^{muz119/120}$	15	280.559	12.870
polg$^{+/119}$	15	454.143	56.471
polg$^{+/+}$	50	340.106	10.066
polg$^{+/121}$	20	403.421	40.916
polg$^{+/120}$	16	362.488	29.611

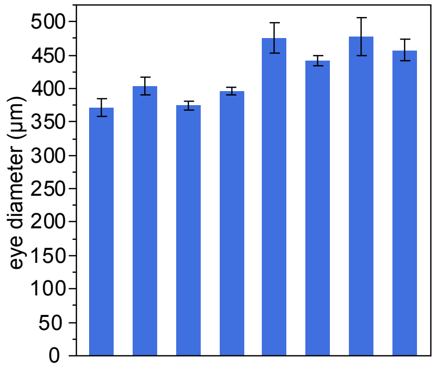

genotype	N	mean	SEM
polg$^{muz120/121}$	3	371.467	3.424
polg$^{muz119/121}$	2	404.300	13.300
polg$^{muz119/119}$	18	374.898	6.300
polg$^{muz119/120}$	18	396.731	5.726
polg$^{+/119}$	27	475.642	22.158
polg$^{+/+}$	60	442.124	7.176
polg$^{+/121}$	21	478.195	28.289
polg$^{+/120}$	18	457.470	16.624

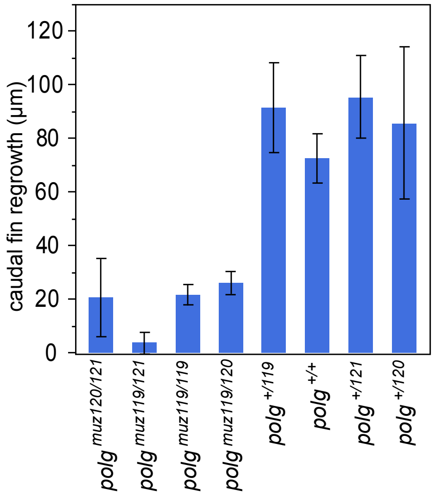

genotype	N	mean	SEM
polg$^{muz120/121}$	2	20.5500	14.450
polg$^{muz119/121}$	2	3.6000	4.200
polg$^{muz119/119}$	17	21.6412	3.964
polg$^{muz119/120}$	12	26.0950	4.128
polg$^{+/119}$	16	91.6613	16.720
polg$^{+/+}$	39	72.5614	9.280
polg$^{+/121}$	16	95.4625	15.514
polg$^{+/120}$	14	85.8260	28.309
